# Associations of Tea Consumption With the Risk of All‐Cause and Cause‐Specific Mortality Among Adults With Type 2 Diabetes: A Prospective Cohort Study in China

**DOI:** 10.1111/1753-0407.70040

**Published:** 2025-01-20

**Authors:** Lifeng Wang, Xikang Fan, Jian Su, Yu Qin, Zhongming Sun, Yan Lu, Shujun Gu, Chong Shen, Jinyi Zhou, Hao Yu, Ming Wu

**Affiliations:** ^1^ Department of Epidemiology and Health Statistics, School of Public Health Southeast University Nanjing China; ^2^ Department of non‐communicable Chronic Disease Control and Prevention Jiangsu Provincial Center for Disease Control and Prevention Nanjing China; ^3^ Department of Chronic Disease Prevention and Control Huai'an City Center for Disease Control and Prevention Huai'an China; ^4^ Department of non‐communicable Chronic Disease Control and Prevention Suzhou Center for Disease Control and Prevention Suzhou China; ^5^ Department of Chronic Disease Prevention and Control Changshu City Center for Disease Control and Prevention Changshu China; ^6^ Department of Epidemiology, School of Public Health Nanjing Medical University Nanjing China

**Keywords:** Chinese, diabetes, mortality, tea

## Abstract

**Aims:**

To investigate the associations of tea consumption with all‐cause and cause‐specific mortality among type 2 diabetes mellitus (T2DM) Chinese patients.

**Materials and Methods:**

The present study included 15 718 participants from the Comprehensive Research on the Prevention and Control of Diabetes between 2013 and 2014 in Jiangsu, China. Information on tea consumption (including frequency, amount, and duration) was collected at baseline using interviewer‐administered questionnaires. Death data were identified by linkage to the Death Certificate System. Cox proportional hazard regression models were used to estimate hazard ratios (HRs) and 95% confidence intervals (CIs).

**Results:**

During a median follow‐up of 9.77 (9.69, 9.82) years, 3046 deaths were documented, including 922 from cardiovascular disease (CVD) and 736 from cancer. Compared with nonconsumers, regular tea consumption (≥ 3 times/week, 1 cup/day, > 30 years) was associated with reduced all‐cause mortality risk in T2DM, with HRs (95% CIs) of 0.82 (0.74, 0.91), 0.80 (0.72, 0.89), and 0.77 (0.68, 0.86). For cardiovascular mortality, the HRs (95% CIs) were 0.79 (0.65, 0.96), 0.72 (0.59, 0.89), and 0.75 (0.60, 0.93). The exposure‐response relationship suggested that consuming 4 g/day may offer the most evident health benefits.

**Conclusions:**

Among Chinese T2DM patients, higher tea frequency and amount intake were associated with lower risk of all‐cause and CVD mortality. It is suggested that consuming 4 g/day of tea could potentially serve as an intervention target. These findings suggest that tea consumption can be a part of a healthy diet for T2DM patients.

## Graphical abstract

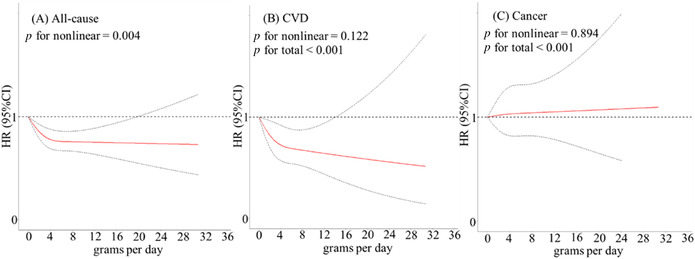

**Summary**

- A prospective cohort study exploring the associations between tea consumption and all‐cause and cause‐specific mortality among Chinese T2DM patients.
- Tea consumption was associated with lower risk of all‐cause and CVD mortality. Consuming 4 g/day of tea could potentially serve as an intervention target.

## 1 Introduction

Diabetes is a chronic disease caused by an absolute or relative insufficiency of insulin secretion and an impairment in its use, and type 2 diabetes mellitus (T2DM) accounted for 90% of all the prevalence [1]. Diabetes was the leading cause of death and disability globally, with nearly 529 million people living with the disease in 2021 [2]. In China, the number of diabetes patients has risen from 90 to 140 million in the past decade [3]. Diabetes and its complications have greatly increased the overall risk of dying prematurely [4].

Tea, the most widely consumed beverage in Asia [5], is prepared by steeping the processed leaves or buds of the *Camellia sinensis* plant in hot water [6]. Its bioactive compounds are known to play a significant role in improving blood sugar control, enhancing insulin activity, and reducing insulin resistance [7]. In the general population, previous studies showed that tea consumption can reduce the risk of mortality [8, 9, 10]. A meta‐analysis of 18 cohort studies found that tea consumption was associated with lower risk of all‐cause and cardiovascular disease (CVD) mortality [11]. Epidemiological evidence based on the Japanese population has also reported a reduced risk of all‐cause and CVD mortality with tea consumption [8, 9, 12]. Furthermore, similar results have been reported among the Chinese population [10, 13]. However, evidence regarding the impact of tea consumption on premature death among patients with T2DM remains scarce. Although a Japanese study reported that tea consumption was inversely associated with all‐cause mortality among T2DM patients [14], overall evidence in this area is still lacking.

Therefore, using the data of 15 718 T2DM patients from the Comprehensive Research on the Prevention and Control of Diabetes (CRPCD) in China, we aimed to investigate the detailed associations of tea consumption with the risk of all‐cause and cause‐specific mortality among Chinese T2DM patients.

## 2 Methods

### 2.1 Study Design and Participants

The data of research participants were derived from a large ongoing population‐based study named “Comprehensive Research on the Prevention and Control of Diabetes” in Jiangsu Province, China. Detailed information about the study has been described previously [15, 16]. Briefly, 44 townships from Jiangsu Province (14 in Suzhou city and 30 in Huai'an city) were selected for the survey by using stratified cluster sampling method. From December 2013 to January 2014, a total of 20 053 T2DM patients who were managed by the National Basic Public Health Service were recruited. T2DM patients were defined as fasting blood glucose levels ≥ 7.0 mmol/L or a self‐report T2DM history without type 1 diabetes. After the participants signed the informed consent form, the trained staff conducted face‐to‐face interviews using standardized questionnaires and standard body measurements. Ethical approval was obtained from the Jiangsu Provincial Center for Disease Control and Prevention (No. 2013026).

In current study, we excluded 4262 participants with cancer, coronary heart disease (CHD), or stroke at baseline to minimize confounding, avoid reverse causality, and enhance the generalizability of the results [13]. Additionally, 87 participants with missing data for tea consumption were further excluded, a total of 15 718 participants were included in the final analysis. When analyzing the various behaviors of tea consumption, the number of participants for each exposure factors was as follows: frequency of tea consumption (*n* = 15 718), consumption grams of tea per day (n = 15 097), consumption cups of tea per day (n = 15 557), and duration of tea consumption (*n* = 15 060) (Figure S1).

### 2.2 Assessment of Tea Consumption

Tea consumption information was assessed for all participants using a baseline questionnaire. (1) Do you have a habit of drinking tea (yes or no)? For those who answered “yes,” we further asked. (2) How many times do you drink tea in a typical week (≥ 3 times/week or 1–2 times/week)? (3) When did you start drinking tea? (4) How much tea leaves do you usually consume each time (with a picture showing the amount of tea leaves in grams)? (5) How many cups of tea do you make per day on average (each time the tea leaves are replaced, it counts as one cup of tea)? (6) Which type of tea do you consume most frequently (e.g., green tea, black tea, oolong tea, or other varieties)? Figure S2 displays images of various tea weights in both their dried and brewed states.

Based on the above question, we divided participants into three groups according to whether they drink tea and frequency of tea consumption (never, 1–2 times/week, ≥ 3 times/week), For drinkers, we further categorized participants according to the consumption grams of tea per day (1–4 g/day, > 4 g/day), consumption cups of tea per day (1 cup/day, ≥ 2 cups/day), and the duration of tea consumption (1–30 years, > 30 years), with reference to previous tea consumption studies [17, 18].

### 2.3 Assessment of Covariates

Covariate information for all participants was collected through baseline questionnaires, including sociodemographic characteristics (age, sex, marital status, education, annual household income), lifestyle behaviors (smoking status, drinking status, physical activity, and intakes of red meat, fresh vegetables, and fruits), and medication use history (insulin, oral antidiabetic medication). Diabetes duration was calculated by subtracting the data of first diagnosis of diabetes from the date of enrolment at the baseline assessment. Information on physical activity was self‐reported by participants using the Global Physical Activity Questionnaire, which has good reliability and validity [19]. To calculate the daily level of physical activity, the metabolic equivalent tasks (METs) value for each type of activity was multiplied by the number of hours spent on each activity, and the total MET‐hours for all activities were summed. Information of fruits, vegetables, and red meat consumption was obtained through food‐frequency questionnaire. Height, weight and waist circumference were measured using uniform equipment. Body mass index (BMI) was calculated as weight (kg) divided by height squared (m^2^).

### 2.4 Assessment of Outcomes

Death information was obtained regularly through the local Death Certificate System. This system provides complete and reliable cause‐specific death information medically certified by doctors [20]. Trained medical staff coded the underlying cause of death by applying the rules from the 10th revision of the International Classification of Diseases (ICD‐10). The primary outcomes were all‐cause mortality, CVD mortality (I00‐I99), and cancer mortality (C00‐C97). In addition, we also separately analyzed major cardiovascular mortality, including CHD (I20‐I25), acute myocardial infarction (I21), stroke (I60‐I61, I63, and I64), hemorrhagic stroke (I60‐I61), and ischemic stroke (I63).

### 2.5 Statistical Analysis

Person‐years were calculated from the date of enrollment to either the date of death or the end of follow‐up (September 30, 2023), whichever came first. Baseline characteristics were calculated based on tea consumption, continuous variables were described by median (interquartile range) and categorical variables were described by proportions (*n*, %). Cox proportional hazards regression models were used to estimate the associations of different tea consumption behaviors with all‐cause and cause‐specific mortality risk by calculating hazard ratios (HRs) and 95% confidence intervals (95% CIs). Two multivariate adjustment models were constructed to account for potential confounding factors. Model 1 was adjusted for age (years), sex (male, female). Model 2 was further adjusted for marital status (married, unmarried), educational level (without formal education, primary and middle school, high school or above, unknown), annual household income (< 40 000 yuan, 40 000–99 999 yuan, ≥ 100 000 yuan, unknown), smoking status (never, previous, current, unknown), alcohol drinking status (never, previous, current, unknown), diabetes duration (years), oral antidiabetic medication use (no, yes), insulin use (no, yes), body mass index (kg/m^2^), total physical activity (MET‐h/day), fruit consumption (never, 1–3 times/week, 4–6 times/week, ≥ 7 times/week, less than weekly), vegetable consumption (never, 1–3 times/week, 4–6 times/week, ≥ 7 times/week, less than weekly), red meat consumption (never, 1–3 times/week, 4–6 times/week, ≥ 7 times/week, less than weekly). Linear trends of all‐cause and cause‐specific mortality related to different tea consumption patterns were calculated by modeling the levels of tea consumption frequency and the amount of tea consumption years and including them as continuous variables in models.

We conducted stratified analyses according to baseline age (< 65, ≥ 65 years), sex (male, female), smoking status (never or previous, current), alcohol drinking status (never or previous, current), and diabetes duration (< 5, ≥ 5 years). The *p* values for interactions were estimated using a likelihood ratio test comparing. Cox proportional risk models with and without cross‐product terms for stratified and exposed variables. We used multivariate cubic regression splines with 4 knots to visually explore nonlinear associations of tea consumption with all‐cause and cause‐specific mortality. A cutoff value was defined as the point where the curve started to level off. In the sensitivity analysis, we first compared the baseline characteristics of participants with (*n* = 19 966) and without (*n* = 15 718) major diseases, including baseline cancer, stroke, and coronary heart disease (CHD). Additionally, we excluded 348 participants who died within the first 2 years of follow‐up, 193 participants who died from accidents (V01‐Y89), 3479 participants with a diabetes duration of less than 1 year, 187 participants who consumed coffee, and 511 participants with baseline kidney disease. We also analyzed data from 19 966 participants, including those with baseline diagnoses of major illnesses such as cancer, coronary heart disease, and stroke. To mitigate potential misclassification of tea consumption data, we also excluded 270 participants categorized under “other” tea types.

All analyses were conducted using the R statistical package, version 4.2.1. *p* values for statistical tests were two‐tailed and significance was defined as *p* < 0.05.

## 3 Results

### 3.1 Baseline Characteristic

A total of 15 718 T2DM participants were included in the study with an average age of 61.98 years. Among them, 5028 participants consumed tea and 3926 participants drank tea ≥ 3 times a week. Compared to nonconsumers, T2DM participants who consumed tea were younger, more likely to be men, with a higher level of education, and drank alcohol (Table 1).

**TABLE 1:** Baseline characteristics of participants according to frequency of tea consumption.

Characteristics	Frequency of tea consumption		
	Never	1–2 times/week	≥ 3 times/week
Participants, *n* (%)	10 690	1102	3926
Age, years	62.33 (9.99)	60.21 (10.80)	61.51 (9.77)
Male, *n* (%)	2263 (21.17)	640 (58.08)	3255 (82.91)
Smoking status, *n* (%)^a^			
Never	7597 (71.07)	809 (73.41)	2831 (72.11)
Previous smoking	622 (5.82)	59 (5.35)	235 (5.99)
Current smoking	2380 (22.26)	229 (20.78)	835 (21.27)
Alcohol drinking status, *n* (%)^a^			
Never	9500 (88.87)	701 (63.61)	1955 (49.80)
Previous drinking	284 (2.66)	83 (7.53)	300 (7.64)
Current drinking	893 (8.35)	317 (28.77)	1657 (42.21)
BMI, kg/m^2^	25.10 (3.48)	25.42 (3.35)	25.37 (3.24)
Marital status, *n* (%)^a^			
Married	1659 (15.52)	104 (9.44)	319 (8.13)
Unmarried	9031 (84.48)	998 (90.56)	3607 (91.87)
Diabetes duration, years	5.59 (5.28)	6.08 (5.47)	6.53 (5.74)
Diabetes medication, *n* (%)			
Oral antidiabetic medication use	7234 (67.67)	773 (70.15)	2757 (70.22)
Insulin use	1365 (12.77)	162 (14.70)	697 (17.75)
Education, *n* (%)^a^			
Without formal education	4942 (46.23)	234 (21.23)	505 (12.86)
Middle school and bellow	5104 (47.75)	688 (62.43)	2754 (70.15)
High school and above	607 (5.68)	177 (16.06)	661 (16.84)
Annual household income, *n* (%), yuan^a^			
≤ 40 000	4727 (44.22)	438 (39.75)	1099 (27.99)
40 000–99 999	4599 (43.02)	512 (46.46)	2022 (51.50)
≥ 100 000	1295 (12.11)	148 (13.43)	788 (20.07)
Total physical activity, MET‐h/day	11.65 (14.60)	13.00 (17.92)	12.07 (18.57)
Diet			
Fruit, *n* (%), times/week			
Never	4014 (37.55)	385 (34.94)	1252 (31.89)
1–3	3441 (32.19)	356 (32.30)	1290 (32.86)
4–6	378 (3.54)	36 (3.27)	166 (4.23)
≥ 7	1327 (12.41)	159 (14.43)	688 (17.52)
Less than weekly	1276 (11.94)	138 (12.52)	398 (10.14)
Vegetable, *n* (%), times/week			
Never	121 (1.13)	4 (0.36)	23 (0.59)
1–3	199 (1.86)	23 (2.09)	58 (1.48)
4–6	126 (1.18)	15 (1.36)	36 (0.92)
≥ 7	9780 (91.49)	1000 (90.74)	3600 (91.70)
Less than weekly	15 (0.14)	3 (0.27)	7 (0.18)
Red meat, *n* (%), times/week			
Never	773 (7.23)	37 (3.36)	109 (2.78)
1–3	5529 (51.72)	531 (48.19)	1796 (45.75)
4–6	993 (9.29)	133 (12.07)	440 (11.21)
≥ 7	2194 (20.52)	301 (27.31)	1218 (31.02)
Less than weekly	920 (8.61)	67 (6.08)	206 (5.25)

Abbreviations: BMI, body mass index; MET, metabolic equivalent tasks.

^a^ For some variables, the totals did not sum to 100% due to small proportions of participants choosing “prefer not to answer.”

### 3.2 Tea Consumption and All‐Cause, Cause‐Specific Mortality

**FIGURE 1:**
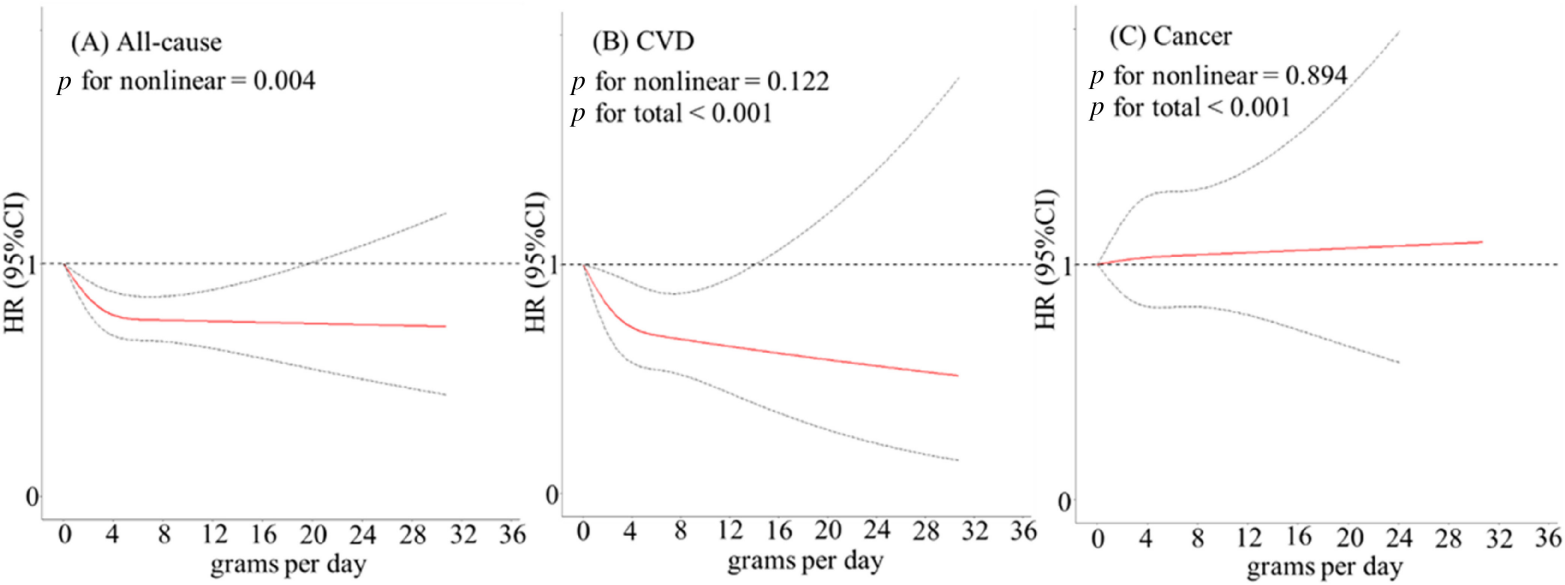
Dose–response relationship between tea consumption with all‐cause (A), CVD (B) and cancer (C) mortality. Results were adjusted for age (years), sex (male, female), educational level (without formal education, primary and middle school, high school or above, unknown), marital status (married, unmarried), annual household income (< 40 000, 40 000–99 999, ≥ 100 000 yuan, unknown), smoking status (never, previous, current, unknown), alcohol drinking status (never, previous, current, unknown), body mass index (kg/m^2^), total physical activity (MET‐h/day), duration of diabetes (years), oral antidiabetic medication use (no, yes), insulin use (no, yes), fruit consumption (never, 1–3 times per week, 4–6 times per week, ≥ 7 times per week, less than weekly), vegetable consumption (never, 1–3 times per week, 4–6 times per week, ≥ 7 times per week, less than weekly), animal meat consumption (never, 1–3 times per week, 4–6 times per week, ≥ 7 times per week, less than weekly).

During a median follow‐up of 9.77 years (interquartile: 9.69–9.82 years), a total of 3046 deaths were recorded, including 922 from CVD and 736 from cancer. Compared with the T2DM population who never drank tea, the fully adjusted HRs (95% CI) for all‐cause mortality were 0.81 (0.73, 0.90) for those who drank tea ≥ 3 times per week, 0.77 (0.67, 0.87) for those who consumed quantity of tea > 4 g per day, 0.85 (0.74, 0.98) for those who consumed quantity of tea ≥ 2 cups per day, and 0.76 (0.68, 0.85) for those whose tea drank duration > 30 years. Similarly, we found that the association of tea consumption with CVD mortality was consistent with all‐cause mortality. Among cancer mortality, we did not find a statistically significant association of tea consumption with cancer mortality (Table 2). In addition, restricted cubic spline analysis showed a nonlinear relationship of the consumption of tea grams per day with all‐cause mortality (*p* for nonlinear = 0.004), and the exposure‐response association kept declining until reaching approximately 4 g per day of tea leaves consumption and then became stabilizing (Figure 1).

**TABLE 2:** HR (95% CI) of all‐cause and cause‐specific mortality according to tea consumption characteristics.

Characteristics	All‐cause mortality (*n* = 3046)			CVD mortality (*n* = 922)			Cancer mortality (*n* = 736)		
	Death/Person year	HR (95%CI)		Death/person year	HR (95% CI)		Death/person year	HR (95% CI)	
		Model 1	Model 2		Model 1	Model 2		Model 1	Model 2
Frequency of tea consumption (times/week)									
Never	2123/95 914	1.00	1.00	654/95 914	1.00	1.00	461/95 914	1.00	1.00
1–2	186/10 022	0.77 (0.66, 0.90)	0.81 (0.69, 0.95)	53/10 022	0.73 (0.55, 0.98)	0.77 (0.57, 1.03)	49/10 022	0.87 (0.64, 1.17)	0.91 (0.67, 1.24)
≥ 3	740/35 449	0.75 (0.68, 0.83)	0.81 (0.73, 0.90)	215/35 449	0.75 (0.63, 0.90)	0.81 (0.67, 0.97)	226/35 449	0.96 (0.79, 1.15)	1.03 (0.84, 1.25)
*p* for trend		< 0.001	< 0.001		0.001	0.022		0.610	0.794
Consumption grams of tea per day (grams/day)									
Never	2123/95 914	1.00	1.00	654/95 914	1.00	1.00	461/95 914	1.00	1.00
1–4	456/21 624	0.78 (0.70, 0.87)	0.84 (0.75, 0.94)	143/21 624	0.81 (0.66, 0.99)	0.86 (0.70, 1.06)	121/21 624	0.90 (0.72, 1.12)	0.95 (0.76, 1.19)
> 4	357/18 168	0.71 (0.62, 0.80)	0.77 (0.67, 0.87)	86/18 168	0.58 (0.45, 0.74)	0.62 (0.48, 0.80)	122/18 168	0.99 (0.79, 1.25)	1.09 (0.85, 1.38)
*p* for trend		< 0.001	< 0.001	< 0.001	< 0.001			0.825	0.587
Consumption cups of tea per day (cups/day)									
Never	2123/95 914	1.00	1.00	654/95 914	1.00	1.00	461/95 914	1.00	1.00
1	618/29 668	0.73 (0.66, 0.81)	0.79 (0.71, 0.88)	174/29 668	0.60 (0.57, 0.83)	0.74 (0.61, 0.90)	174/29 668	0.88 (0.72, 1.07)	0.95 (0.78, 1.17)
≥ 2	277/14 369	0.81 (0.70, 0.92)	0.85 (0.74, 0.98)	82/14 369	0.84 (0.65, 1.07)	0.87 (0.68, 1.13)	93/14 369	1.07 (0.84, 1.37)	1.11 (0.86, 1.43)
*p* for trend		< 0.001	< 0.001		0.008	0.054		0.957	0.564
Duration of tea consumption (years)									
Never	2123/95 914	1.00	1.00	654/95 914	1.00	1.00	461/95 914	1.00	1.00
1–30	260/19 576	0.79 (0.69, 0.90)	0.81 (0.71, 0.93)	70/19 576	0.75 (0.58, 0.97)	0.76 (0.59, 0.99)	75/19 576	0.87 (0.67, 1.12)	0.90 (0.69, 1.17)
> 30	519/20 071	0.70 (0.62, 0.78)	0.76 (0.68, 0.85)	151/20 071	0.68 (0.55, 0.84)	0.73 (0.59, 0.91)	157/20 071	0.92 (0.74, 1.14)	1.01 (0.81, 1.26)
*p* for trend		< 0.001	< 0.001		< 0.001	0.003		0.387	0.997

*Note:* Model 1 was adjusted for age (years), sex (male, female). Model 2 was further adjusted for educational level (without formal education, primary and middle school, high school or above, unknown), marital status (in marriage, not in marriage), annual household income (< 40 000, 40 000–99 999, ≥ 100 000 yuan, unknown), smoking status (never, previous, current, unknown), alcohol drinking status (never, previous, current, unknown), body mass index (kg/m^2^), total physical activity (MET‐h/day), duration of diabetes (years), oral antidiabetic medication use (no, yes), insulin use (no, yes), fruit consumption (never, 1–3 times per week, 4–6 times per week, ≥ 7 times per week, less than weekly), vegetable consumption (never, 1–3 times per week, 4–6 times per week, ≥ 7 times per week, less than weekly), animal meat consumption (never, 1–3 times per week, 4–6 times per week, ≥ 7 times per week, less than weekly).

### 3.3 Subgroup Analysis and Sensitivity Analysis

As shown in Table 3 and Table 4, tea consumption was inversely associated with the risk of stroke mortality, but not statistically significant in other subtypes of CVD. Compared with T2DM participants who never drank tea, those who drank tea ≥ 3 times per week, consumed tea > 4 g per day, consumed cups of tea 1 per day, drank duration of tea > 30 years had 28% (HR = 0.72, 95% CI: 0.53–0.97), 48% (HR = 0.52, 95% CI: 0.35–0.79), 33% (HR = 0.67, 95% CI: 0.49–0.91), and 37% (HR = 0.63, 95% CI: 0.45–0.89) lower stroke mortality risk, respectively (Table 3).

**TABLE 3:** HR (95% CI) of CVD subtypes mortality according to tea consumption characteristics.

Characteristics	CHD mortality (*n* = 159)			Acute myocardial infarction mortality (*n* = 99)			Stroke mortality (*n* = 394)		
	Death/Person year	HR (95%CI)		Death/Person year	HR (95%CI)		Death/Person year	HR (95%CI)	
		Model 1	Model 2		Model 1	Model 2		Model 1	Model 2
Frequency of tea consumption (times/week)									
Never	103/95 914	1.00	1.00	69/95 914	1.00	1.00	290/95 914	1.00	1.00
1–2	11/10 022	0.97 (0.51, 1.85)	0.93 (0.48, 1.78)	5/10 022	0.71 (0.28, 1.79)	0.68 (0.27, 1.76)	23/10 022	0.71 (0.46, 1.09)	0.78 (0.50, 1.22)
≥ 3	45/35 449	1.00 (0.66, 1.53)	1.01 (0.65, 1.56)	25/35 449	0.91 (0.53, 1.58)	0.93 (0.53, 1.65)	81/35 449	0.62 (0.46, 0.82)	0.72 (0.53, 0.97)
*p* for trend		0.987	0.976		0.706	0.782		< 0.001	0.026
Consumption grams of tea per day (grams/day)									
Never	103/95 914	1.00	1.00	69/95 914	1.00	1.00	290/95 914	1.00	1.00
1–4	26/21 624	0.91 (0.56, 1.46)	0.92 (0.57, 1.50)	14/21 624	0.82 (0.44, 1.54)	0.84 (0.44, 1.60)	60/21 624	0.76 (0.56, 1.03)	0.86 (0.63, 1.17)
> 4	22/18 168	0.91 (0.54, 1.54)	0.93 (0.54, 1.61)	13/18 168	0.91 (0.46, 1.80)	0.95 (0.47, 1.93)	31/18 168	0.45 (0.30, 0.68)	0.52 (0.35, 0.79)
*p* for trend		0.695	0.757		0.698	0.809		< 0.001	0.003
Consumption cups of tea per day (cups/day)									
Never	103/95 914	1.00	1.00	69/95 914	1.00	1.00	290/95 914	1.00	1.00
1	41/29 668	1.05 (0.69, 1.59)	1.02 (0.66, 1.58)	20/29 668	0.83 (0.47, 1.47)	0.83 (0.46, 1.49)	64/29 668	0.57 (0.42, 0.77)	0.67 (0.49, 0.91)
≥ 2	14/14 369	0.93 (0.51, 1.71)	0.98 (0.53, 1.82)	10/14 369	1.06 (0.51, 2.21)	1.11 (0.52, 2.34)	36/14 369	0.79 (0.54, 1.16)	0.86 (0.59, 1.27)
*p* for trend		0.913	0.979		0.920	0.996		0.017	0.132
Duration of tea consumption (years)									
Never	103/95 914	1.00	1.00	69/95 914	1.00	1.00	290/95 914	1.00	1.00
1–30	12/19 576	0.87 (0.46, 1.62)	0.82 (0.44, 1.55)	5/19 576	0.56 (0.22, 1.43)	0.56 (0.22, 1.43)	29/19 576	0.66 (0.44, 0.98)	0.71 (0.47, 1.06)
> 30	34/20 071	1.03 (0.64, 1.64)	1.03 (0.63, 1.68)	20/20 071	1.06 (0.57, 1.94)	1.07 (0.56, 2.02)	55/20 071	0.55 (0.40, 0.76)	0.63 (0.45, 0.89)
*p* for trend		0.949	0.968		0.998	0.979		< 0.001	0.006

*Note:* Model 1 was adjusted for age (years), sex (male, female). Model 2 was further adjusted for educational level (without formal education, primary and middle school, high school or above, unknown), marital status (married, unmarried), annual household income (< 40 000, 40 000–99 999, ≥ 100 000 yuan, unknown), smoking status (never, previous, current, unknown), alcohol drinking status (never, previous, current, unknown), body mass index (kg/m^2^), total physical activity (MET‐h/day), duration of diabetes (years), oral antidiabetic medication use (no, yes), insulin use (no, yes), fruit consumption (never, 1–3 times per week, 4–6 times per week, ≥ 7 times per week, less than weekly), vegetable consumption (never, 1–3 times per week, 4–6 times per week, ≥ 7 times per week, less than weekly), animal meat consumption (never, 1–3 times per week, 4–6 times per week, ≥ 7 times per week, less than weekly).

**TABLE 4:** HR (95% CI) of stroke subtypes mortality according to tea consumption characteristics.

Characteristics	Hemorrhagic stroke mortality (*n* = 145)			Ischemic stroke mortality (*n* = 199)		
	Death/Person year	HR (95%CI)		Death/Person year	HR (95%CI)	
		Model 1	Model 2		Model 1	Model 2
Frequency of tea consumption (times/week)						
Never	99/95 914	1.00	1.00	159/95 914	1.00	1.00
1–2	9/10 022	0.71 (0.35, 1.42)	0.77 (0.38, 1.57)	12/10 022	0.72 (0.39, 1.31)	0.81 (0.44, 1.50)
≥ 3	37/35 449	0.68 (0.44, 1.05)	0.80 (0.51, 1.27)	28/35 449	0.43 (0.27, 0.68)	0.51 (0.32, 0.81)
*p* for trend		0.245	0.679		< 0.001	0.005
Consumption grams of tea per day (grams/day)						
Never	99/95 914	1.00	1.00	159/95 914	1.00	1.00
1–4	25/21 624	0.78 (0.48, 1.25)	0.88 (0.54, 1.43)	27/21 624	0.68 (0.44, 1.07)	0.78 (0.50, 1.24)
> 4	14/18 168	0.47 (0.25, 0.85)	0.55 (0.30, 1.03)	9/18 168	0.28 (0.14, 0.57)	0.33 (0.16, 0.68)
*p* for trend		0.050	0.192		< 0.001	0.003
Consumption cups of tea per day (cups/day)						
Never	99/95 914	1.00	1.00	159/95 914	1.00	1.00
1	27/29 668	0.59 (0.37, 0.95)	0.70 (0.43, 1.15)	25/29 668	0.44 (0.28, 0.71)	0.54 (0.33, 0.86)
≥ 2	16/14 369	0.79 (0.44, 1.41)	0.87 (0.49, 1.57)	14/14 369	0.65 (0.36, 1.17)	0.70 (0.39, 1.28)
*p* for trend		0.401	0.768		0.009	0.052
Duration of tea consumption (years)						
Never	99/95 914	1.00	1.00	159/95 914	1.00	1.00
1–30	15/19 576	0.74 (0.41, 1.31)	0.81 (0.46, 1.46)	12/19 576	0.58 (0.32, 1.07)	0.62 (0.34, 1.16)
> 30	24/20 071	0.58 (0.35, 0.96)	0.68 (0.40, 1.14)	20/20 071	0.41 (0.24, 0.69)	0.48 (0.28, 0.83)
*p* for trend		0.115	0.336		< 0.001	0.005

*Note:* Model 1 was adjusted for age (years), sex (male, female). Model 2 was further adjusted for educational level (without formal education, primary and middle school, high school or above, unknown), marital status (married, unmarried), annual household income (< 40 000, 40 000–99 999, ≥ 100 000 yuan, unknown), smoking status (never, previous, current, unknown), alcohol drinking status (never, previous, current, unknown), body mass index (kg/m^2^), total physical activity (MET‐h/day), duration of diabetes (years), oral antidiabetic medication use (no, yes), insulin use (no, yes), fruit consumption (never, 1–3 times per week, 4–6 times per week, ≥ 7 times per week, less than weekly), vegetable consumption (never, 1–3 times per week, 4–6 times per week, ≥ 7 times per week, less than weekly), animal meat consumption (never, 1–3 times per week, 4–6 times per week, ≥ 7 times per week, less than weekly).

We further analyzed the relationship of tea consumption with all‐cause mortality in specific population subgroups. The strength of the associations of tea consumption with the risk of stroke was largely consistent across subgroups, when classified by age, sex, smoking status, alcohol drinking status, and diabetes duration (*p* for interaction > 0.05) (Table S1). In the sensitivity analysis, we first compared the baseline characteristics of participants with (*n* = 19 966) and without (*n* = 15 718) major diseases, including baseline cancer, stroke, and coronary heart disease (CHD). The comparison revealed that participants with baseline major diseases had higher age, BMI, and diabetes duration, and lower physical activity levels (Table S2). Excluding participants who died within the first 2 years of follow‐up (*n* = 348), had diabetes duration ≤ 1 year (*n* = 3479), died from accidents (*n* = 193), consumed coffee (*n* = 187), or had baseline kidney disease (*n* = 511) did not substantially affect the findings (Table S3). The analysis of data from 19 966 participants, including those with baseline diagnoses of major illnesses, showed that the association between tea consumption and the risk of all‐cause mortality, CVD mortality, and cancer mortality was consistent with the primary outcomes (Table S4). Participants classified as “other” tea types were excluded, resulting in findings that remained consistent with the primary results (Table S5).

## 4 Discussion

In this ongoing prospective cohort with a relatively large sample size, 91.48% of participants who consumed tea preferred green tea. We primarily assessed tea consumption based on weekly frequency, as this measure is both accessible to participants and reflective of their habitual tea‐drinking patterns. We found that tea consumption was associated with reduced mortality risk due to all‐cause, CVD, and stroke in the T2DM population.

Although other metrics, such as grams per day and cups per day, were considered, weekly frequency was prioritized as the primary measure due to its reliability in capturing participants' habitual intake. Regular tea consumption (≥ 3 times per week) was linked to a reduced mortality risk, with additional analyses indicating that individuals consuming more than 4 g of tea per day or 1cup per day might experience greater health benefits. Restricted cubic spline analysis revealed a nonlinear relationship between tea consumption and all‐cause mortality, indicating that a daily intake of 4 g of tea may serve as a potential intervention target for individuals with T2DM.

In the general population, previous studies have shown that tea consumption was inversely associated with the risk of all‐cause and CVD mortality [11, 21]. A large prospective cohort study in UK Biobank showed that higher tea consumption was associated with lower all‐cause mortality [22]. Among adults with T2DM, few studies have been conducted to investigate the association between tea consumption and mortality. One large cohort study based on the health professionals in the United States showed that higher tea and coffee consumption was associated with decreased all‐cause mortality in patients with T2DM [23]. In the current study, we found inverse associations of tea consumption with mortality risk due to all‐cause and CVD in the Chinese T2DM population. Furthermore, many prospective epidemiological studies have investigated the dose–response relationship between tea consumption and major causes of death in the general population, but the observed J‐shaped and U‐shaped associations remain controversial [24, 25]. A meta‐analysis included 856 206 individuals showed U‐shaped associations between tea consumption and all‐cause mortality [24], whereas, the UK Biobank study revealed a reverse J‐shaped relationship between tea consumption and all‐cause mortality, the lowest risk of mortality was associated with a threshold consumption of approximately 3 cups per day of tea [25]. The observed J‐shaped association between tea consumption and all‐cause mortality in our study aligns with findings from the UK Biobank study [25], indicating that a daily intake of 1–4 g or more than 4 g similarly lowers mortality risk, with 4 g marking the inflection point in the dose–response curve. This suggests that a daily tea consumption of 4 g may serve as a potential intervention target for individuals with T2DM.

With respect to tea consumption with CVD mortality, the negative association found in our study is consistent with some prior observational studies and meta‐analysis in the general population [9, 17, 24, 26]. The Japan Public Health Center‐based Prospective Study indicated a negative correlation between tea intake and mortality rates from a variety of causes, including heart disease and cerebrovascular disease [9]. The America Multi‐Ethnic Study of Atherosclerosis found that regular tea drinkers had significantly lower incidence of CVD events when compared with never drinkers [27]. However, the Tehran Lipid and Glucose Study found that those in the highest tertile of tea consumption had elevated risk of CVD mortality [28]. A previous meta‐analysis of tea consumption in relation to CVD concluded that the geographic region where the studies were conducted appeared to explain much of the heterogeneity in the results for tea consumption and CVD outcomes [29]. Black tea is the most consumed type of tea in Iran, often containing a variety of additives or artificial colors, and it is also common for black tea to be accompanied by sweets or sugar [30]. Our study analyzed the main types of CVD among T2DM and found that tea consumption might lower the risk of stroke and ischemic stroke mortality, but not for CHD mortality. In line with our study, a recent meta‐analysis of prospective studies, involving 513 804 participants, found that an increase in tea consumption was associated with decreased risk of stroke [31]. However, for the outcome of CHD, a meta‐analysis of 24 studies showed that increased tea consumption was associated with a lower risk of death from CHD [24]. The inconsistency may be explained by the lower number of patients with coronary heart disease in our study.

The polyphenols, rich in tea, are known to alleviate the risk of CVD by various mechanisms, including anti‐inflammatory, anti‐oxidant or anti‐thrombotic properties of phenolic acids, as well as endothelial improvement and platelet aggregation inhibition [32, 33, 34]. In addition, a 12‐week randomized clinical trial suggested that supplementary intake of tea extract may improve arterial stiffness in patients with T2DM [35]. Meanwhile, tea consumption may also reduce the risk of cardiovascular death by lowering blood glucose levels and CPR concentrations and improving insulin resistance in patients with T2DM [36].

In the current study, no association was found between tea consumption and cancer mortality among T2DM. The evidence of consuming tea on cancer mortality is lacking among T2DM and inconsistent in the general population. Cohort studies from Japanese and Northern Manhattan showed that tea was inversely associated with risk of total cancer mortality [37, 38]. In contrast, the Japanese Osaki Study, the Shanghai Men's Health Study, and the Shanghai Women's Health Study demonstrated that tea consumption was not associated with cancer mortality [10, 12]. This inconsistency may be due to the lower mortality rate from cancer in the Japanese Study and the higher prevalence of smoking in Shanghai.

The present analysis has several strengths. First, the primary strength of our study was that the study population was drawn from a Chinese community‐based diabetes cohort with a larger sample size. Second, patients with pre‐existing illnesses were excluded to minimize reverse causation. Third, the mortality monitoring system can ensure us to complete long‐term follow‐up for both all‐cause and CVD mortality. However, several limitations need to be considered. First, recall bias was inevitable, as the information of tea consumption was collected from participants through questionnaires. Second, tea consumption has been self‐reported by participants, which may not reflect long‐term consumption patterns. Third, as this study was observational, there may still be potential reverse causality and residual confounding.

## 5 Conclusion

In conclusion, our research adds to the evidence suggesting that tea consumption is inversely associated with all‐cause and CVD mortality in the T2DM population, especially stroke mortality. Regular consumption of tea and drinking 4 g of tea per day may yield greater health benefits. Therefore, further causal inference studies, such as randomized controlled trials are needed to validate our findings.

## Author Contributions

M.W. and H.Y. contributed to the conception and design of the study. J.S., Y.Q., Y.L., Z.S., and S.G. contributed to the data collection. L.W. and X.F. have full access to all the data in the study and take responsibility for the integrity of the data and the accuracy of the data analysis. L.W., X.F., J.S., and H.Y. did the statistical analysis and drafted the manuscript. Y.Q., Z.S., Y.L., S.G., C.S., and J.Z. critically revised the manuscript for important intellectual content. M.W. had primary responsibility for the final content. All authors reviewed and approved the final manuscript. The corresponding authors attest that all listed authors meet authorship criteria and that no others meeting the criteria have been omitted.

## Conflicts of Interest

The authors declare no conflicts of interest.

## Supporting information


Data S1.
