# Supplementary material for: Associations of Tea Consumption With the Risk of All‐Cause and Cause‐Specific Mortality Among Adults With Type 2 Diabetes: A Prospective Cohort Study in China
Source: J Diabetes. 2025 Jan 20;17(1):e70040. doi: 10.1111/1753-0407.70040 (PMC11744464; doi:10.1111/1753-0407.70040)
Supplement: Supplementary file 1 — Data S1. [file JDB-17-e70040-s001.zip › BRA2020090.pdf]

中共江苏省委组织部
江苏省人才工作领导小组办公室
江苏省科学技术厅

苏人才办〔2020〕9号

关于下达2020年度省第五期“333工程”科研项目资助计划的通知

各设区市市委组织部、市人才工作领导小组办公室、市科学技术局，省有关部门：

经专家评审、省“333工程”专家委员会审定，2020年度省第五期“333工程”科研项目资助计划已确定。所资助项目将列入省级科技计划项目管理，项目编号见附件。现将资助经费计划下达给你们。

本次共资助科研项目432项，资助经费总额为4760万元。所资助项目实施期限一般为2年。根据《江苏省第五期“333高层次人才培养工程”专项资金管理办法》（苏财规〔2016〕17号）规定，项目资助经费首次拨付70%，项目完成经考核验收通过后

拨付30%。请资助对象所在单位严格安排匹配经费，加强经费管理，确保专款专用。获得资助的培养对象要按照项目计划进度，积极做好科研工作。各设区市、省有关部门每年向省人才办报告一次项目进展情况，及时督促培养对象保质保量地完成项目并按时提交结题报告。

请通知获得资助的培养对象抓紧签订《省第五期“333工程”培养资金资助项目合同书》（一式四份）。各设区市人才办、省有关部门认真审核项目资助合同书并盖章确认后，将其中一份于2020年8月10日之前报省人才办备案。

省委组织部人才工作处联系人：刘怡萱，电话：025—83395366、83393812（传真）；省科技厅政策法规处联系人：陈利琴，电话：025—86505159。

附件：1．2020年度省第五期“333工程”科研资助立项项目

2．省第五期“333工程”培养资金资助项目合同书

中共江苏省委组织部　　　江苏省人才工作领导小组办公室

江苏省科学技术厅

2020年7月　日

序号	姓　名	工作单位及职务	项目名称	培养层次	资助金额(万元)	申报单位	项目编号
80	董自波	江苏海洋大学研究员	三类医疗器械—肠镜清肠消泡剂的研制	二	25	省教育厅	BRA2020080
81	刘波	苏州科技大学研究员	Cr 掺杂 SbTe 光电混合存储材料及其超快相变机理研究	二	30	省教育厅	BRA2020081
82	汪仁	江苏省中国科学院植物研究所研究室主任	石蒜咖啡酸氧甲基转移酶 1 提高汞离子胁迫抗性的分子机制研究	二	30	省科技厅	BRA2020082
83	许剑冰	国网电力科学研究院有限公司系统保护实验室副主任	交直流电网系统保护安全稳定控制系统级实验验证关键技术研究	二	30	省科技厅	BRA2020083
84	李群	国网江苏省电力有限公司电力科学研究院副院长	电网移相器关键技术研究及应用	二	30	省工信厅	BRA2020084
85	李忠辉	中国电子科技集团公司第五十五研究所首席专家	面向 5G 移动通讯的 4 英寸 GaNHEMT 外延材料批产技术研究	二	20	省工信厅	BRA2020085
86	杨桂新	江苏省交通运输厅科技处处长	钢结构桥梁防腐涂层寿命评估及维修新技术研究	二	25	省交通厅	BRA2020086
87	李中华	南京水利科学研究院水工所研究室副主任	基于 AI 技术的船闸水动力安全风险识别和预警	二	30	省水利厅	BRA2020087
88	王宗志	南京水利科学研究院水文所研究室主任	大规模调水扰动的梯级湖泊水文效应与安全调控研究	二	35	省水利厅	BRA2020088
89	嵇富海	苏州大学附属第一医院麻醉手术科主任	miR-499a-5p 调控靶基因 FOXO4 对心肌缺血再灌注损伤线粒体凋亡的作用及其机制研究	二	25	省卫生健康委	BRA2020089
90	武鸣	江苏省疾病预防控制中心主任	基于社区队列的老年 2 型糖尿病并发症预测及强化干预研究	二	35	省卫生健康委	BRA2020090
91	喻春钊	南京医科大学第二附属医院科技处处长	肿瘤代谢在结直肠癌肝转移治疗中的应用探索	二	30	省卫生健康委	BRA2020091
92	朱雪松	苏州大学附属第一医院科技处副处长（主持工作）、临床研究院办公室主任	N-乙酰半胱氨酸生物活性骨水泥促进椎体骨缺损修复的机制研究	二	30	省卫生健康委	BRA2020092

附件 2

省第五期“333 工程”培养资金资助项目合同书

经专家评审，省人才工作领导小组批准，2020 年度省第五期“333 工程”培养资金资助 江苏省疾病预防控制中心 （单位）武鸣 同志主持的项目 基于社区队列的老年 2 型糖尿病并发症预测及强化干预研究 （名称） 研究经费 35 万元。资助对象必须于 2022 年 7 月前完成所获资助项目的研究及成果转化工作。项目完成后，及时将有关结果报省人才办备案。资助对象所在单位必须严格执行江苏省“333 高层次人才培养工程”培养资金管理办法的规定，专款专用，并安排匹配经费。对资助对象及其所在单位不符合规定的经费开支，以及无正当理由终止合同或未能如期完成项目的，省人才办视情况追回部分或全部经费。

本合同一式 4 份，省人才办、市或省有关主管部门、资助对象及所在单位各 1 份。

资助对象（签字）武鸣

所在单位（盖章）

2020 年 7 月 30 日

市或有关主管部门（盖章）

2020 年 8 月 30 日

附件1

2020年度省第五期“333高层次人才培养工程”科研资助项目申报书

申报人姓名：武　鸣

工作单位：江苏省疾病预防控制中心

所属一级学科：公共卫生与预防医学

归属设区市（部门）：江苏省卫生健康委员会

单位所在县（市、区）：南京市鼓楼区

江苏省人才工作领导小组办公室制

二〇一九年

一、申报简表

<table>
<tr><td rowspan="4">研究项目</td><td>名　　称</td><td colspan="8">基于社区队列的老年2型糖尿病并发症预测及强化干预研究</td></tr>
<tr><td>所属学科</td><td colspan="2">公共卫生</td><td>项目类型</td><td colspan="3">A. 基础研究　B.应用研究</td><td>B</td></tr>
<tr><td>项目来源</td><td colspan="6">A.国家级项目　B.部、省级项目　C.市、厅级项目　D.个人自选项目</td><td>D</td></tr>
<tr><td>申报金额</td><td>50</td><td>万元</td><td>起止年月</td><td colspan="4">2020年　12月　至　2022年　12月</td></tr>
<tr><td rowspan="8">申报人简况</td><td>姓　　名</td><td>武鸣</td><td>出生年月</td><td>1974年7月</td><td colspan="2">“333工程”培养层次</td><td colspan="2">第二</td></tr>
<tr><td>性　　别</td><td>男</td><td colspan="2">身份证号（护照号码）</td><td colspan="4">342324197407070078</td></tr>
<tr><td>民　　族</td><td>汉</td><td>学　　历</td><td colspan="2">研究生</td><td>学　位</td><td colspan="2">博士</td></tr>
<tr><td colspan="2">最终学历获得单位与时间</td><td colspan="6">南京铁道医学院1999.7</td></tr>
<tr><td>现从事专业</td><td colspan="2">慢性非传染病及伤害预防控制</td><td colspan="2">专业技术职务</td><td colspan="3">主任医师</td></tr>
<tr><td>所在单位</td><td colspan="2">江苏省疾病预防控制中心</td><td colspan="2">归属设区市（部门）</td><td colspan="3">江苏省卫生健康委员会</td></tr>
<tr><td>通讯地址</td><td colspan="2">南京市江苏路172号</td><td colspan="2">邮政编码</td><td colspan="3">210009</td></tr>
<tr><td>办公室电话</td><td colspan="2">025-83759981</td><td colspan="2">手机号码</td><td colspan="3">13701402423</td></tr>
<tr><td rowspan="2">项目组</td><td>总人数</td><td>高级职称</td><td>中级职称</td><td>初级职称</td><td>博士生</td><td>硕士生</td><td>本科生</td><td>参加单位数</td></tr>
<tr><td>7</td><td>4</td><td>3</td><td></td><td></td><td></td><td></td><td></td></tr>
</table>

研究内容和意义	摘要	1.内容：<br>1.1 糖尿病患者综合防治队列建立。以基本公共卫生服务项目社区糖尿病患者健康管理为基础，在常熟市、淮安市淮安区、清江浦区建立的 2 万名糖尿病患者队列基础上，进行为期 3 年的随访干预研究。<br>1.2 老年 2 型糖尿病并发症的影响因素分析与预测预警模型建立。连续动态收集患者人口学资料，生活方式，用药情况，以及生化检测指标，系统评价血糖控制效果，糖尿病并发症的发生情况及其环境、行为和遗传影响因素，建立老年 2 型糖尿病患者并发症的影响因素与预测模型，研发适宜推广使用的糖尿病并发症风险评估工具。<br>2 意义：<br>2.1 社区糖尿病强化管理模式的建立。在社区统一管理基础上，开展老年糖尿病患者强化管理研究，对研究对象进行基于互联网的、以社区规范化管理、专家指导、社区健康教育、病人自我管理相结合的糖尿病强化管理研究，探讨建立可推广的、经济可行的糖尿病并发症社区强化管理模式。<br>2.1 通过队列研究，研发适宜的老年 2 型糖尿病并发症风险评估模型和评估工具，开展并发症风险评估，识别风险较高人群，及时开展筛查、早期诊断并治疗和干预。<br>2.2 形成可推广应用的糖尿病并发症社区糖尿病强化管理模式，从而降低致残率和致死率，解决糖尿病预防、控制和管理的瓶颈问题，切实提高江苏省糖尿病的防治水平。<br>2.3 通过对糖尿病并发症社区强化管理模式进行成本-效益等卫生经济学评价，形成可推广的循证学证据。

项目成员：覃玉

学历

2002/11-2012/10，荷兰瓦赫宁根大学，人类营养学系，博士

1997/09-2000/06，南京医科大学，流行病与统计学系，硕士

1990/09-1995/06，江西医学院，预防医学系，学士

工作简历

2000/08-至今，江苏省疾病预防控制中心慢性非传染性疾病防制所，现为主任医师

1995/08-1997/08，江西省九江县卫生防疫站卫生科，医师

论著

1、张伟伟，吴同浩，马进，董建梅，李伟伟，秦绪成，马昭君，苏健，崔岚，周金意，**覃玉**. 江苏省中老年 35-75 岁人群超重/肥胖现状及其危险因素分析. 预防医学,2019；31(9):886-891.（通讯作者）

2、缪伟刚，**覃玉**，苏健，崔岚，罗鹏飞，杜文聪，周金意. 江苏省不同血糖水平人群心血管病危险因素聚集分析.中华疾病控制杂志,2019:23(4):436-440.（通讯作者）

3、管芳，**覃玉**，苏健，吕淑荣，潘晓群，陶然，周金意，武鸣. 江苏省 18~69 岁男性自报慢性病与戒烟行为关系. 中华疾病控制杂志, 2019;10(10):1230-1234.（通讯作者）

4、**Qin Yu**, Peian Lou, Peipei Chen, Lei Zhang, Pan Zhang, Guiqiu Chang, Ning Zhang, Ting Li, Cheng Qiao. Interaction of poor sleep quality, family history of type 2 diabetes, and abdominal obesity on impaired fasting glucose:a population-based cross-sectional survey in China. Int J Diabetes Dev Ctries. 2015 online DOI 10.1007/S13410-015-0410-4. (第一作者)

5、Lou Peian, **Qin Yu**, Zhang Pan, Chen Peipei, Zhang Lei, Chang Guiqiu, Li Ting, Qiao Cheng, Zhang Ning. Association of sleep quality and quality of life in type 2 diabetes mellitus: a cross-sectional study in China. Diabetes Res Clin Pract. 2015;107:69-76. (通讯作者)

项目成员：苏健

学历

2015-09 至 2018-07，东南大学，公共卫生学院，硕士，导师：武鸣

2004-09 至 2009-07，南京医科大学，公共卫生学院，学士

工作简历

2016-01 至现在，江苏省疾病预防控制中心，慢性非传染性疾病防制所，主管医师

2009-08 至 2015-12，江苏省疾病预防控制中心，慢性非传染性疾病防制所，医师

论著

苏健，覃玉，潘晓群，沈冲，高艳，潘恩春，张永青，周金意，武鸣*. 新鲜水果摄入与 2 型糖尿病患者血糖控制关系的研究，中华流行病学杂志, 2019, 40(6):

660-665.

苏健，吕淑荣，杨婕，陶然，覃玉，周金意，张永青，武鸣*. 江苏省成人脂质蓄积指数与高血压和糖尿病患病风险关系的研究，中华疾病控制杂志, 2018, 22(3): 217-221,271.

苏健，覃玉，沈冲，高艳，潘恩春，杜文聪，周金意，张永青，武鸣*. 江苏省社区管理 2 型糖尿病患者综合控制情况分析，中华内分泌代谢杂志, 2018, 34(2): 112-120.

苏健，覃玉，沈冲，高艳，潘恩春，潘晓群，陶然，张永青，武鸣*. 吸烟和戒烟行为与男性 2 型糖尿病血糖控制关系的研究，中华流行病学杂志, 2017, 38(11): 1454-1459.

苏健，陶然，周金意，杨婕，覃玉，胡一河，陆艳，金建荣，卞铮，郭彧，陈铮鸣，李立明*，武鸣*. 成年人睡眠状况与糖尿病患病关系的研究，中华流行病学杂志, 2017, 38(5): 597-60.

项目成员：朱政

学历

2006/09-2009/06　复旦大学/上海市肿瘤研究所，上海医学院病原生物学系，博士，导师：顾健人

2001/09-2004/06　中国药科大学，生物制药学院微生物与生化药学系，硕士，导师：刘景晶

1997/09-2001/06　中国药科大学，生物制药学院微生物制药系，学士

工作简历

2015/07-至今，江苏省疾病预防控制中心，慢性非传染病防制所，副研究员

2009/08-2015/06，江苏省疾病预防控制中心，病原微生物研究所，助理研究员

论著

1． **Zhu Z#***; Li Y#; Zhou J; Zhang Y; Cao RY*; Acute enterovirus infections significantly alter host cellular DNA methylation status, Infect Genet Evol, 2020, 80: 104190

2．Chen F#; **Zhu Z#**; van Duijnhoven FJB; Dong M; Qian Y; Yu H; Yang J; Cui L; Han R; Su J; Du W; Zhou J; Wu M*; Genetic Variants in Group-Specific Component (GC) Gene Are Associated with Breast Cancer Risk among Chinese Women, Biomed Res Int , 2019, 2019: 3295781.

3．**Zhu Z#**; Teng Z#; van Duijnhoven FJB; Dong M; Qian Y; Yu H; Yang J; Han R; Su J; Du W; Huang X; Zhou J; Yu X; Kampman E; Wu M*; Interactions between RASA2, CADM1, HIF1AN gene polymorphisms and body fatness with breast cancer: a population-based case-control study in China, Oncotarget, 2017, 8(58): 98258-269

4．**Zhu Z***; Qi Y; Fan H; Cui L; Shi Z; Systematic Identification and Bioinformatic Analysis of MicroRNAs in Response to Infections of Coxsackievirus A16 and Enterovirus 71. Biomed Res Int, 2016, 2016: 4302470.

5．**Zhu Z#**; Hao X#; Yan M; Yao M; Ge C; Gu J; Li J*; Cancer stem/progenitor cells
