# Supplementary material for: Associations of Tea Consumption With the Risk of All‐Cause and Cause‐Specific Mortality Among Adults With Type 2 Diabetes: A Prospective Cohort Study in China
Source: J Diabetes. 2025 Jan 20;17(1):e70040. doi: 10.1111/1753-0407.70040 (PMC11744464; doi:10.1111/1753-0407.70040)
Supplement: Supplementary file 1 — Data S1. [file JDB-17-e70040-s001.zip › H2023022.pdf]

江苏省疾病预防控制中心合同档案
编号：JSCDC-KY-202300292

密级：不涉密

江苏省卫生健康委科研项目合同书

课题编号：H2023022

课题名称：基于脂代谢和生活方式评分的糖尿病合并心脑血管疾病风险预测研究

主持部门：江苏省卫生健康委员会

保证单位：江苏省疾病预防控制中心（江苏省公共卫生研究院）

承担单位：江苏省疾病预防控制中心（江苏省公共卫生研究院）

地址：南京市江苏路172号　　邮政编码：210009

项目负责人：范习康 单位电话：025-83759415 手机：18915999608

电子邮箱：xikangfan1991@jscdc.cn

起止日期：2024-01-01至2026-12-31

结题时间：2027-12-31

江苏省卫生健康委员会制

二零二二年

一、主要研究内容和预期的技术指标：

主要研究内容：

1. 系统分析糖尿病并发心脑血管疾病的脂代谢特征并筛选潜在预警标志物

基于江苏省社区糖尿病队列，采用巢式病例对照研究，以常见心脑血管疾病（脑卒中、冠心病）为结局，分别选取确诊病例50例，并按性别、年龄和居住地进行1:2匹配糖尿病对照，构成本研究的发现集。使用研究对象基线的血浆，采用UPLC Ultimate 3000色谱串联Q-Exactive质谱技术平台靶向检测12种脂代谢物（甘油三酯、总胆固醇、极低密度脂蛋白胆固醇、低密度脂蛋白胆固醇、高密度脂蛋白胆固醇、鞘磷脂、花生四烯酸、亚油酸、乙酰乙酸、β-羟基丁酸、丙酮、磷脂酰胆碱）。完成原始数据质控后，利用定性及定量标化分析定库，采用采用正交偏最小二乘判别分析（Orthogonal Partial Least Square-Discriminate Analysis，OPLS-DA）筛选具有显著统计学意义的物质，系统鉴定具有预测潜力的特征脂代谢物。

2. 基于特征脂代谢物和生活方式评分构建风险预测模型

基于第一阶段鉴定的糖尿病血浆特征代谢物，将糖尿病队列中剩余的大样本人群作为验证集。以常见CVD作为结局，选择所有确诊患者，以未发CVD的糖尿病者作为对照。通过基线分析筛选出研究对象心脑血管疾病风险相关变量（社会人口学特征、生活习惯、膳食模式、疾病史、家族史等），参考现有评分系统构建生活方式评分，采用Cox比例风险回归构建糖尿病合并CVD的风险预测模型。采用逐步渐进法依次带入特征代谢物，并进一步分析代谢物与生活方式评分的交互作用。通过计算受试者工作特征曲线（Receiver Operation Characteristic Curve，ROC）及曲线下面积（Area Under Curve，AUC）、净重新分类指数（Net Reclassification Improvement，NRI）等指标评价模型预测效能。

关键技术：

1. 靶向脂代谢组学技术

使用超高效液相色谱串联质谱技术靶向检测糖尿病患者基线血浆脂代谢物水平。靶向代谢组学能够较准确的反映出人体当前状态，能够精确定量检测特定脂代谢通路的目标产物，并且检测价格较低，代价较小，

性价比较高，便于常态化检测的推广。

2. 生活方式评分构建技术

采用赋分法对每个生活方式进行量化。采用吸烟、饮酒、锻炼、膳食、BMI 及睡眠等 6 项指标定义生活方式是否健康。达到健康标准则记为 1 分，反之为 0 分。总分范围为 0-6 分，评分越高代表生活方式越健康。

3. 基于真实世界的糖尿病并发心脑血管疾病的风险预测模型构建技术

基于江苏省现有的社区糖尿病队列人群，通过分析糖尿病患者基线生活方式和脂代谢物水平，使用 Cox 回归模型完成模型构建。利用逐步回归保证模型的稳健性，通过 ROC 曲线下面积（AUC）、净重新分类指数（Net Reclassification Improvement, NRI）等指标评价模型的预测能力。

预期的技术指标：

1. 完成基于血浆脂代谢和基于生活方式评分的糖尿病合并心脑血管疾病风险预测模型的构建和评价。

2. 形成项目专题报告 1 份，发表学术论文 2-4 篇，其中 SCI 论文 1-2 篇。

3. 培养硕士研究生 1-2 名。

正式稿

二、合同期内的研究进度计划，分年度达到的目标和研究方法及技术路线，包括时间进度安排、研究地点、规模（参加人数、经费投入），阶段成果，所采取的主要方法和技术路线：

研究方法

1. 探索糖尿病患者并发心脑血管疾病的脂代谢特征

1.1 研究对象

采用巢式病例对照研究设计，于江苏省社区糖尿病队列人群中选择研究对象。该队列于 2013 年 12 月-2014 年 1 月启动，采用整群随机抽样方法在常熟市、淮安市清河区和淮安区的 65 个乡镇/街道中抽取 44 个乡镇/街道。以已纳入基本公共卫生服务的 35 岁以上的 2 型糖尿病患者为研究对象，共计 20340 名 2 型糖尿病患者参与现场调查，随后排除身体状况较差、精神状态异常不能接受调查，或不愿意参与调查者，最终纳入研究 20053 名，城市和农村人口比例为 1:2。本次基线调查建立了完整的基线流行病学数据库和生物标本库。所有研究对象从基线入组后开始随访。截至 2021 年 12 月 31 日，共发现确诊心脑血管疾病 2633 例。

分别选取确诊心脑血管疾病病例 50 例，并按性别、年龄和居住地进行 1:2 匹配糖尿病对照。本研究中，首先排除患有严重肝肾疾病及恶性肿瘤者、因身体原因无法参与调查者、患有精神疾患或拒绝调查者。病例组纳入标准为：经二级及以上医院诊断的，具有临床或影像学确诊报告（CT 或 MRI）的新发糖尿病合并心脑血管疾病患者。糖尿病对照组为新发 2 型糖尿病患者，且未发生血栓、动脉粥样硬化、脑卒中等心脑血管疾病；血糖正常组为健康人群，未被确诊为糖尿病或血脂异常。对照组人群均通过临床诊断排除患有心脑血管疾病的可能。

1.2 资料和样本收集

（1）问卷调查

采用《社区糖尿病综合干预及应用项目调查问卷》进行面对面调查，主要包括基本信息、生活行为方式（吸烟、饮酒、膳食、运动、睡眠质量等）、家族史、主要病史及控制情况（脑卒中、心脏病、高血压、糖尿病、血脂异常）等。

（2）体格测量

研究对象的体格检查包括身高、体重、腰围、颈围和血压测量以及

心脏和颈动脉听诊。由专业人员采用心脏听诊判定项目对象是否心律整齐。

（3）疾病诊断

由经验丰富的专业心血管内科、超声科医生对项目对象中的高危人群进行颈动脉超声、头部 CT 以及冠状动脉造影检查。收集病例组和对照组临床检查报告，明确病例的确诊信息。

（4）生物样本的采集

调查对象在完成调查问卷后，经培训合格的医院工作人员使用抗凝采血管采集其空腹静脉血样本，于现场立即放入低速离心机完成离心（4000r/min，离心 8 分钟），并完成血浆标本的分装并按照问卷 ID 号进行编码。离心后的血浆样本需在冷藏环境 2-8℃下保存，完成编码后存入-80℃冰箱储存。

1.3 血浆靶向脂代谢组学检测

（1）样本预处理

将待测血浆样本于 4℃条件下解冻。吸取样本 100μL 置于 1.5mL 离心管中，加入三倍体积预冷有机溶剂，涡旋 5min，于冰上静置 10min 后，在 4℃下 12000rpm 离心 10min，取上清液置于离心管中，氮气吹干。溶剂采用分析级甲醇或乙腈，加入 100μL 进行复溶，涡旋 5min，冰上静置 10min 后，再次完成离心，并取上清液进样分析。

（2）色谱质谱条件

色谱条件：色谱柱：Waters UPLC BEH C18 毛细管柱（2.1×100mm，1.7um），柱温 45℃，样品室 4℃。流动相为 0.2%甲酸水溶液/甲醇进行梯度洗脱，进样量：2.0μL，流速：0.4mL/min。

质谱条件：采用 Xevo G2-S Q-TOF 串联三重四级杆飞行时间质谱系统。离子源为电喷雾离子源（ESI）；扫描方式：正、负离子模式，喷雾电压为 3.0kV，锥孔电压为 25V，离子源温度为 120℃，锥孔气流速为 50L/h，使用高纯氮气作为辅助喷雾电离与脱溶剂气体，干燥气流速为 10mL/min，雾化气温度正离子模式下为 450℃，负离子模式下为 400℃，MSe 模式下检测。

（3）数据预处理

将通过 UPLC-Q/TOF-MS 分析方法获得的质谱数据（.raw 格式文件）导入 Progenesis QI 数据分析软件，完成保留时间矫正、峰识别、峰提取、基线校正、峰积分、峰对齐等数据前处理过程。获得校正后 Excel 格式的二维数据矩阵，包括质荷比（m/z），保留时间和归一化峰面积。

（4）多元统计分析

在 MetaboAnalyst5.0 在线分析软件中，采用正交偏最小二乘判别分析（Orthogonal Partial Least Square-Discriminate Analysis, OPLS-DA）建模来筛选对于区分各组样本贡献较大的差异代谢物。OPLS-DA 模型得分图中评价模型质量的参数有 R2X（模型对 X 矩阵的解释率），R2Y（模型对 Y 矩阵的解释率）和 Q2Y（模型的预测能力）。其中，R2 和 Q2 值越接近于 1 说明该模型越稳定、可靠。一般情况下 R2 和 Q2 值>0.5 即可证明所建模型质量较高。此外，OPLS-DA 建模事先对检测样本进行了分组，所以为保证模型可靠性，需采用响应置换检验（Response permutation test）和 10 倍循环交叉验证（10-fold Cross validation）来判断和验证建模过程是否存在过拟合现象，以防止发生过拟合。通过 OPLS-DA 模型生成变量权重重要性排序（Variable importance in projection, VIP）并采用 t 检验。VIP>1.0 和 P<0.05 的代谢物可以作为区分不同对照组的差异代谢物。根据差异代谢物进行代谢通路分析；根据文献调研和 ROC Curve 分析筛选特征代谢标志物用于后续验证。

2. 基于特征脂代谢物和生活方式评分进行风险预测模型构建

2.1 研究对象

与第一部分不同，进行建模时，研究对象拓展至社区糖尿病队列所有人群作为验证集。病例为新发的所有心脑血管疾病患者，以未发病的糖尿病者作为对照。研究对象的纳入和排除标准与第一部分相同。

2.2 代谢物靶向人群验证

选取验证集人群基线血浆标本，对鉴定发现的特征靶向脂代谢物进行检测。检测技术和统计方法与第一阶段保持一致，检测物质为第一阶段筛选出的具有差异性表达的特征代谢物。采用 Cox 比例风险模型，调整基线一般人口学信息、生活习惯、膳食模式以及疾病史、家族史等混杂因素，分析每一种特征代谢物与缺血性卒中发病风险间是否存在时序性关联。验证特征代谢物表达水平和缺血性卒中发病风险间的关联与第一阶段研究结果是否一致，从而进一步鉴定缺血性脑卒中发病的特征性代谢物。

2.3 生活方式评分构建

对于研究对象的多种生活方式因素，采用赋分法对每个因素进行量化。采用吸烟、饮酒、锻炼、膳食、BMI 及睡眠等 6 项指标定义生活方式是否健康。满足以下标准则定义为健康：(1)当前不吸烟，包括从不吸烟或者戒烟；(2)适度饮酒：酒精摄入量男性每天＞0-＜25 g 或女性每天＞0-＜15 g；(3)适度锻炼：7-＜10 h /周；(4)均衡膳食，至少满

足其中4项：每天吃蔬菜、每天吃水果、每周吃红肉1-6d、每周吃豆类≥4 d、每周吃鱼≥1 d 和经常饮茶；(5)维持正常体重：18.5-＜24 kg /m2；(6)适宜的睡眠时长：7-8h/晚。达到健康标准则记为1分，反之为0分。总分范围为 0-6 分，评分越高代表生活方式越健康。将研究对象分为五组：0-1、2、3、4和>4，以0-1分组为参照组。

2.4糖尿病合并心脑血管疾病发病风险预测模型的构建

基于鉴定的糖尿病血浆特征脂代谢物，采用Cox回归联合生活方式评分，综合一般人口学特征（如年龄、性别、经济水平、教育程度等）以及医药史（个人患病、卒中家族史、药物治疗史等）多方面因素，构建心脑血管发病风险预测模型。通过计算受试者工作特征曲线（Receiver Operation Characteristic Curve,ROC)及曲线下面积(Area Under Curve，AUC）、净重新分类指数（Net Reclassification Improvement，NRI）、决策曲线净获益值（Net benefit）等指标评价模型预测效能。

进度安排

本项目研究期限为3年，自2024年1月至2026年12月。具体计划如下：

（1）2024年1月-2024年12月：

于江苏省常熟市、淮安市清河区和淮安区收集基线问卷资料和血液标本，探索糖尿病患者并发心脑血管疾病的脂代谢特征；开展血浆靶向脂代谢组学检测、筛选代谢物；整理队列随访数据，为人群验证集做准备。

（2）2025年1月-2025年12月：

完成生活方式评分方法的构建与计算；确定验证的研究对象和血液样本情况，完成验证队列研究对象血浆样本的靶向代谢物检测；初步构建基于特征脂代谢物和生活方式评分的风险预测模型。

（3）2026年1月-2026年6月：

优化风险预测模型参数，验证模型效能并评价人群应用价值。完成基于血浆脂代谢和基于生活方式评分的糖尿病合并心脑血管疾病风险预测模型的构建和评价。

（4）2026年7月-2026年12月：

总结数据、撰写并发表相应学术论文2-4篇，其中SCI论文1-2篇，形成项目专题报告1份，完成项目结题工作，培养硕士研究生1-2名。

人员规模

本项目团队由8人组成，研究员、副教授、副主任医师、主管医师以及在读博士各1名，在读研究生3名；预计总经费10万元，其中省级财政资助额度5万，课题承担单位自筹5万元。具体分配方式如下：

（1）2024年1月-2024年7月：

基线问卷资料质控及血液标本整理送测工作，参与人数5人，涉及劳务费0.5万元；基线资料及生物样本质量以及代谢检测方案可行性论证工作，参与人数3人，涉及费用包括：资料费0.2万元，差旅/会议费0.5万元，专家咨询费0.25万元；本阶段合计费用1.45万元。

（2）2024年8月-2024年12月：

完成发现队列血浆靶向脂代谢组学检测、筛选代谢物，参与人数4人，涉及费用包括：靶向代谢组学发现集检测费1万元，出版/文献/知识产权事务费0.4万元，劳务费0.6万元；本阶段合计费用2万元。

（3）2025年1月-2025年12月：

完成验证队列血浆靶向脂代谢组学检测，结合生活方式评分初步构建风险预测模型，参与人数6人，涉及费用包括：代谢组学验证集检测费2.6万元，劳务费0.8万元，出版/文献/知识产权事务费0.4万元，本阶段合计费用3.8万元。

（4）2026年1月-2026年12月：

优化风险预测模型参数，验证模型效能并评价人群应用价值，总结数据、撰写并发表相应学术论文，参与人员2人，涉及费用包括：资料费0.2万元，差旅/会议费0.5万元，专家咨询费0.25万元，劳务费0.3万元，出版/文献/知识产权事务费1.5万元，本阶段合计费用2.75万元。

正式稿

三、预期达到的技术经济效益和社会效益：

1. 预期的技术经济效益

（1）高水平代谢组学检测及预测模型构建技术：a）利用社区糖尿病队列发现糖尿病合并CVD的重要暴露因素，定量估计生活方式和脂代谢对CVD发病的影响；利用风险预测模型，指导糖尿病患者的精准治分类管理措施的确立，为糖尿病和心脑血管疾病的防治政策提供科学依据。b）通过开展慢性病风险评估和筛查，精准预测高风险人群，提高糖尿病患者生活质量。评价模型的预测效能，科学评估高危人群管理和干预措施的有效性和经济性，便于社会资源的合理分配和推广应用。

（2）推动糖尿病及心脑血管疾病的精准预防：a）通过宣传推广项目成果，在糖尿病人群中坚持开展科学、规范的健康宣教，增加公众对高质量医学证据的理解。增加本地居民对人群医学研究的关注度及对科学研究过程和方法的理解，提高今后研究的参与配合度。b)为全省相关领域研究提供更高质量的科研平台，产生更多高水平的研究成果，提升我省学者在国际学术舞台上的地位和影响力，增加平等交流与合作的机会，进一步吸引更多途径、更大力度的科研投入。

2. 预期的社会效益

（1）促进江苏省糖尿病及心脑血管疾病精准防治：在社区糖尿病队列的基础上，更加精准地评估糖尿病合并心脑血管疾病的发病风险。通过多种生活方式进行组合并评分，寻找具有特征性的危险因素，并采用代谢组学鉴定特征脂代谢物，提高慢性病防治的工作精度和预测能力。

（2）新型生物标志物的发现与推广：经过实验室检测和统计分析发现糖尿病合并心脑血管疾病风险预测模型中的特征代谢物，作为反映人体暴露的特征物质，并进一步推广到临床用于早期诊断，将其纳入常规检测指标。

（3）推动糖尿病及心脑血管疾病风险预测领域的创新：整合脂代谢组学以及宏观生活方式，构建糖尿病并发心脑血管疾病发病风险预测模型，促进代谢通路及传统病因学等学术领域的融会贯通。

四、项目承担单位和参加单位研究分工情况，请按排列顺序列出（注：“经费分配”是指项目总经费的分配，单位为万元）：

单位名称	研究分工	经费分配	主研人员签字
江苏省疾病预防控制中心（江苏省公共卫生研究院）	负责项目的总体设计，组织培训，指导现场实施，质量控制，采集糖尿病及并发疾病数据，构建基于脂代谢和生活方式的心脑血管疾病风险模型。	8	范习康<br>范习康
南京医科大学	负责代谢组学技术指导、数据分析，协助构建多组学分析模型和生活方式评分系统。	2	杭栋<br>杭栋

正式稿

五、甲方确认在合同规定的研究期间拨付给乙方研究经费如下：

单位：万元

研究经费总　额	省卫生健康委拨款	其它部门拨款	自筹	贷款	其它
10	5	0	5	0	0

如有其它部门拨款，请在下面按要求说明。

拨款单位名称	拨款总额（万元）	拨款时间	其它说明

六、各类型项目须按照省财政资助额度,项目承担单位给予不低于1:1比例的资金配套。项目经费须专款专用，按合同中的预算开支,不得挪用或截留，如发生此类情况，一经查实，甲方有权终止项目;并追回所拨项目经费。

七、在项目实施过程中，甲方主要负责组织协调解决重大问题。根据合同执行情况，必要时在专家论证的基础上，与丙方协商对合同进行调整。乙方负责组织完成合同所规定的各项研究任务，丙方负责监督检查，保证合同的实施。各方应保证合同中计划经费的投入。

八、在项目实施过程中，如乙方根据研究情况要对合同进行修改，需及时向甲方或丙方书面提出，经甲、丙方组织讨论同意后方有效，并按修改后的合同执行。乙方自行修改无效，并负责赔偿所有损失。

九、合同期满后，由甲方组织专家进行统一结题，未按合同要求完成者，取消日后申请委科研项目资格，同时甲方有权追回拨款。

十、验收后的项目，如需进行临床试验的要在有关部门批准后方可进行。

十一、签约各方对秘密资料负有保密责任，未经甲方批准，不得在公开发表的论文中引用保密数据，试验结果或其它有关资料，也不得泄露给甲、乙、丙三方之外的单位和个人。

十二、本合同必须经过项目主持部门（甲方）—江苏省卫生健康委员会、项目承担单位（乙方）、项目保证单位（丙方）—当地卫生健康委员会三方共同签字盖章才有效。

十三、本合同正式文本一式四份，甲方留存一份，乙、丙方及负责人各存一份。

正式稿

十四、签约各方：

主持部门（甲方）：江苏省卫生健康委员会<br><br>主管处室负责人（签章）：叶荣<br>年　月　日
承担单位（乙方）：江苏省疾病预防控制中心（江苏省公共卫生研究院）<br><br>开户银行：民生银行南京凤凰西街支行　帐号：0802014210002340<br><br>开户名：江苏省疾病预防控制中心（江苏省公共卫生研究院）<br><br>法定代表人或委托代理人（签章）：[signature]　　项目负责人（签章）：[signature]<br><br>2023年12月22日年　月　日
保证单位（丙方）：江苏省疾病预防控制中心（江苏省公共卫生研究院）<br><br>法定代表人或委托代理人（签章）：[signature]<br>2023年12月22日

附件信息

附件名称
5 科研合作协议
JSJK2023-B033-01 伦理批件

正式稿

科研合作协议

甲方：江苏省疾病预防控制中心（江苏省公共卫生研究院）

乙方：南京医科大学

本协议双方就共同申报江苏省卫生健康委 2023 年度医学科研项目——面上项目，项目名称为：基于脂代谢和生活方式评分的糖尿病合并心脑血管疾病风险预测研究。经友好协商，依据有关法规和计划委托单位的有关要求，达成如下协议，并共同遵守本协议涉及的全部内容。

一、任务分工与责任

甲方（项目申报单位）：负责项目的总体设计，组织培训，指导现场实施，质量控制，采集糖尿病及并发疾病数据，构建基于脂代谢和生活方式的心脑血管疾病风险模型。

乙方（项目合作单位）：负责代谢组学技术指导、数据分析，协助构建多组学分析模型和生活方式评分系统。

二、经费分配

项目获得资助后，甲、乙双方按课题研究整体需要分配省资助经费。

三、成果分配

本项目研究所产生的成果及产权归属，由双方商定。任何一方发表研究论文，需标注项目名称及编号，署名由双方协商。

四、数据保密

甲、乙双方有义务对全部数据资料进行保密，且仅可将数据用于双方科研工作，不得外泄给任何第三方或用作商业用途。不得使用该数据从事危害国家安全、社会公共利益和他人合法利益的活动，如违反上述规定并构成犯罪的，由司法机关追究其责任。

五、其他

1.协议经甲、乙双方法定代表人或委托代理人签字并盖章后生效；

2.项目获批，则协议有效期自动延伸至结题验收为止。项目未获批准，则协议自动终止。

3.本合同未尽事宜，甲、乙双方本着互惠互利，友好协商原则另行约定，并以备忘录或附件形式体现。

4.本协议一式两份，各执壹份，具有同等法律效力。

甲方：江苏省疾病预防控制中心

（江苏省公共卫生研究院）

签字：[illegible]

盖章：江苏省疾病预防控制中心（江苏省公共卫生研究院） 3201061206032

日期：[illegible]

乙方：南京医科大学

签字：[illegible]

盖章：南京医科大学 科技合同专用章

日期：

江苏省疾病预防控制中心伦理审查委员会

Ethics Committee of Jiangsu Provincial Center for Disease Prevention and Control

伦理审查批件

Ethics Review Approval

<table>
<tr><td>批件号</td><td colspan="3">JSJK2023-B033-01</td></tr>
<tr><td>项目名称</td><td colspan="3">基于脂代谢和生活方式评分的糖尿病合并心脑血管疾病风险预测研究</td></tr>
<tr><td>项目来源</td><td colspan="3">江苏省卫生健康委科研项目</td></tr>
<tr><td>研究单位</td><td colspan="3">江苏省疾病预防控制中心（江苏省公共卫生研究院）</td></tr>
<tr><td>主要研究者</td><td colspan="3">范习康</td></tr>
<tr><td>审查类别</td><td>初始审查</td><td>审查方式</td><td>快速审查</td></tr>
<tr><td>审查日期</td><td>2023 年 12 月 21 日</td><td>审查地点</td><td>委员所在地</td></tr>
<tr><td>审查委员</td><td colspan="3">翟祥军，李雷</td></tr>
<tr><td colspan="4">批准文件</td></tr>
<tr><td colspan="4">1、研究方案（版本号：V1.0，版本日期：2023 年 05 月 09 日）
2、知情同意书（版本号：V1.0，版本日期：2023 年 05 月 09 日）</td></tr>
<tr><td colspan="4">审查文件</td></tr>
<tr><td colspan="4">1、初始审查申请
2、主要研究者简历
3、研究团队成员信息
4、研究经费来源说明
5、生物样本、信息数据的来源说明（版本号：V1.0，版本日期：2023 年 05 月 09 日）
6、关于公布江苏省卫生健康委 2023 年度医学科研立项项目的通知（苏卫科教[2023]11 号）
7、研究材料诚信承诺书
8、无利益冲突申明
9、研究成果的发布形式说明</td></tr>
<tr><td colspan="4">审查意见</td></tr>
<tr><td colspan="4">根据国家卫生健康委、教育部、科技部和中医药局《涉及人的生命科学和医学研究伦理审查办法》、原国家卫生与计划生育委员会《涉及人的生物医学研究伦理审查办法》以及 WMA《赫尔辛基宣言》和 CIOMS《涉及人的健康相关研究国际伦理指南》的伦理原则，经本伦理审查委员会审查，同意按所批准的研究方案、知情同意书开展本项研究。
研究过程中若变更主要研究者，对研究方案、知情同意书、招募材料等的任何修改，请提交修正案审查申请。
如申请人暂停或提前终止研究，请及时提交暂停/终止研究报告。
请于 2024-12-21 前 1 个月内提交研究进展的首次跟踪审查报告（跟踪审查频率：12 个月/次）。
完成研究，请申请人提交结题报告。</td></tr>
<tr><td colspan="4">说明：本批件自批准之日起一年内有效（2023-12-22 ~ 2024-12-21），逾期未实施，自行废止。</td></tr>
</table>

联系人	钱姣	联系电话	025-83759513
主任委员（或被授权者）签字	[illegible]	日期（XXXX 年 XX 月 XX 日）	2023 年 12 月 22 日
江苏省疾病预防控制中心伦理审查委员会（盖章）			

江苏省疾病预防控制中心伦理审查委员会
