# Supplementary material for: Associations of Tea Consumption With the Risk of All‐Cause and Cause‐Specific Mortality Among Adults With Type 2 Diabetes: A Prospective Cohort Study in China
Source: J Diabetes. 2025 Jan 20;17(1):e70040. doi: 10.1111/1753-0407.70040 (PMC11744464; doi:10.1111/1753-0407.70040)
Supplement: Supplementary file 1 — Data S1. [file JDB-17-e70040-s001.zip › jdb70040-sup-0001-Supinfo.docx]

**Associations of tea consumption with the risk of all-cause and cause-specific mortality among adults with type 2 diabetes: A prospective cohort study in China**

Lifeng Wang^1†^ Xikang Fan^2†^, Jian Su^2^, Yu Qin^2^, Zhongming Sun^3^, Yan Lu^4^, Shujun Gu^5^, Chong Shen^6^, Jinyi Zhou^2^, Hao Yu^2‡^ Ming Wu^1,2‡^

^†^ Contributed equally as first authors

^‡^ Contributed equally as joint corresponding authors.

**Online Supplementary Material**

**Supplementary Figure 1.** Flowchart of study participants from the CRPCD project.

**Supplementary Figure 2.** Images of various tea weights in dried (A) and brewed (B) states.

**Supplement Table 1.** Association between tea consumption and all-cause mortality risk stratified by potential risk factors.

**Supplement Table 2.** Participants were based on baseline characteristics before and after the exclusion of significant disease.

**Supplement Table 3.** Association between tea consumption and all-cause mortality risk stratified by potential risk factors.

**Supplement Table 4.** HR (95% CI) of all-cause and cause-specific mortality according to tea consumption characteristics(n = 19 966).

**Supplement Table 5.** HR (95% CI) of all-cause and cause-specific mortality according to tea consumption characteristics(n = 15 448).


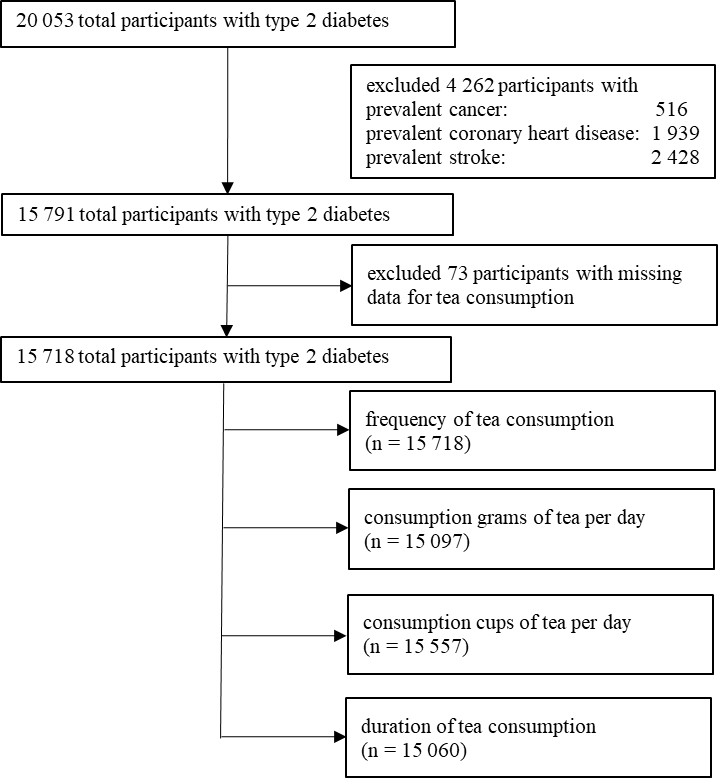


Supplementary Figure 1. Flowchart of study participants from the CRPCD project.


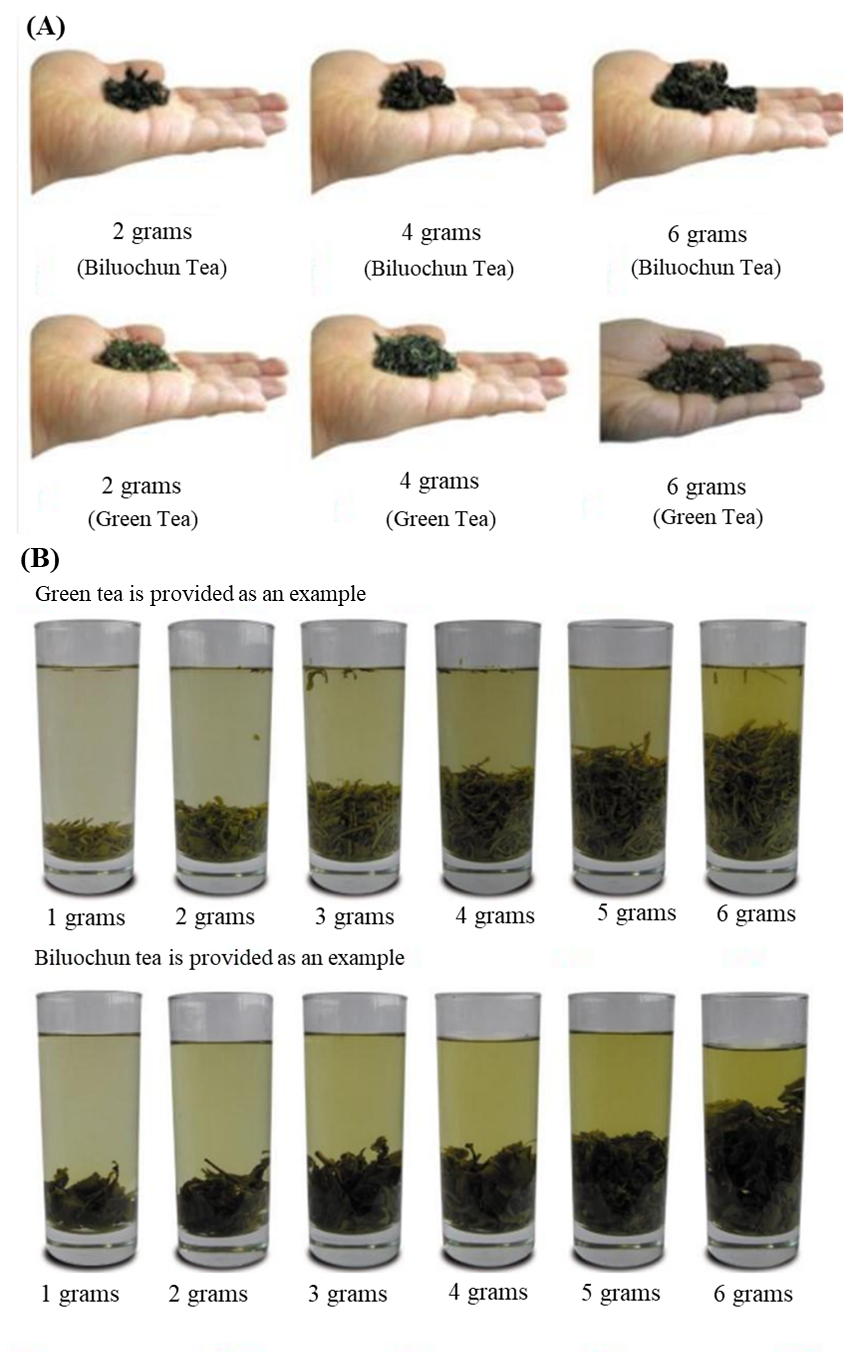


Supplementary Figure 2. Images of various tea weights in dried (A) and brewed (B) states.

Biluochun Tea (also known as "Green Snail Spring" due to its curled, snail-like shape) is one of China’s most famous green teas. Originating from Dongting Mountain in Jiangsu Province, it is renowned for its delicate, spiraled leaves, floral fragrance, and a rich, refreshing taste. The tea is harvested in early spring and is highly prized for its tender young leaves and buds. Green Tea is a type of tea made from the leaves of the Camellia sinensis plant. Unlike black tea, green tea is minimally oxidized during processing, which helps retain its green color and high levels of antioxidants. It is widely consumed around the world, known for its light, fresh taste and health benefits.

| Supplement Table 1. Association between tea consumption and all-cause mortality risk stratified by potential risk factors | | | | | |
| --- | --- | --- | --- | --- | --- |
| Subgroup | No. of all-cause mortality | Frequency of tea consumption (times /week) | | | *P* for interaction |
|  |  | Never | 1-2 | ≥3 |  |
| Age (years) | |  |  |  | 0.141 |
| <65 | 808 | 1.00 | 0.85 ( 0.64 , 1.13 ) | 0.86 ( 0.72 , 1.04 ) |  |
| ≥65 | 2 241 | 1.00 | 0.78 ( 0.65 , 0.94 ) | 0.80 ( 0.71 , 0.91 ) |  |
| Sex |  |  |  |  | 0.525 |
| Male | 1 375 | 1.00 | 0.78 ( 0.65 , 0.95 ) | 0.80 ( 0.71 , 0.90 ) |  |
| Female | 1 674 | 1.00 | 0.86 ( 0.65 , 1.12 ) | 0.88 ( 0.71 , 1.09 ) |  |
| Smoking status |  |  |  |  | 0.643 |
| Never or previous | 2 331 | 1.00 | 0.78 ( 0.65 , 0.93 ) | 0.82 ( 0.73 , 0.92 ) |  |
| Current | 690 | 1.00 | 0.91 ( 0.66 , 1.25 ) | 0.80 ( 0.64 , 1.00 ) |  |
| Drinking status |  |  |  |  | 0.443 |
| Never or previous | 2 542 | 1.00 | 0.77 ( 0.64 , 0.93 ) | 0.79 ( 0.70 , 0.89 ) |  |
| Current | 505 | 1.00 | 0.93 ( 0.68 , 1.28 ) | 0.92 ( 0.75 , 1.13 ) |  |
| Diabetes duration (years) | | |  |  | 0.112 |
| <5 | 1 374 | 1.00 | 1.00 ( 0.79 , 1.28 ) | 0.84 ( 0.72 , 0.98 ) |  |
| ≥5 | 1 675 | 1.00 | 0.71 ( 0.57 , 0.87 ) | 0.79 ( 0.68 , 0.90 ) |  |
| HRs were calculated in Cox proportional hazards model. Model1 was adjusted for age (years), sex (male, female). Model 2 was further adjusted for educational level (without formal education, primary and middle school, high school or above, unknown), marital status(married, unmarried),annual household income (< 40 000, 40 000-99 999, ≥ 100 000 yuan, unknown), smoking status (never, previous, current, unknown), alcohol drinking status (never, previous, current, unknown), body mass index (kg/m^2^), total physical activity (MET-h/day), duration of diabetes (years), oral antidiabetic medication use (no, yes), insulin use (no, yes),fruit consumption (never, 1-3 times per week, 4-6 times per week, ≥ 7 times per week, less than weekly), vegetable consumption (never, 1-3 times per week, 4-6 times per week, ≥ 7 times per week, less than weekly), animal meat consumption (never, 1-3 times per week, 4-6 times per week, ≥ 7 times per week, less than weekly). | | | | | |

| Supplement Table 2. Participants were based on baseline characteristics before and after the exclusion of significant disease | | | |
| --- | --- | --- | --- |
| Characteristics | With Significant Disease | Without Significant Disease | *P* |
| Participants, n (%) | 19 966 | 15 718 |  |
| Frequency of tea consumption (times/week) | |  |  |
| Never | 13705 | 10690 | 0.444 |
| 1-2 | 1368 | 1102 |  |
| ≥3 | 4893 | 3926 |  |
| Age, years | 62.87 (9.86) | 61.98 (10.01) | <0.001 |
| Male, n (%) | 7 845 (39.29) | 6 158 (39.18) | 0.836 |
| Smoking status, n (%)^a^ |  |  |  |
| Never | 14 303 (71.64) | 11 237 (71.49) | 0.872 |
| Previous smoking | 1 132 ( 5.67) | 916 ( 5.83) |  |
| Current smoking | 4 387 (21.97) | 3 444 (21.91) |  |
| Alcohol drinking status, n (%)^a^ |  |  |  |
| Never | 15 560 (77.93) | 12 156 (77.34) | <0.001 |
| Previous drinking | 983 ( 4.92) | 667 ( 4.24) |  |
| Current drinking | 3 387 (16.96) | 2 867 (18.24) |  |
| BMI, kg/m^2^ | 25.34 (3.47) | 25.19 (3.42) | <0.001 |
| Marital status, n (%)^a^ |  |  |  |
| Married | 2 759 (13.82) | 2 082 (13.25) | 0.121 |
| Unmarried | 17 207 (86.18) | 13 636 (86.75) |  |
| Diabetes duration, years | 6.10 (5.66) | 5.86 (5.43) | <0.001 |
| Diabetes medication, n (%) |  |  |  |
| Oral antidiabetic medication use | 13 823 (69.23) | 10 764 (68.48) | 0.296 |
| Insulin use | 3 018 (15.12) | 2 224 (14.15) | 0.034 |
| Education, n(%)a |  |  |  |
| Without formal education | 7 403 (37.08) | 5 681 (36.14) | 0.179 |
| Middle school and bellow | 10 638 (53.28) | 8 546 (54.37) |  |
| High school and above | 1 856 ( 9.30) | 1 445 ( 9.19) |  |
| Annual household income, n (%), yuan |  |  |  |
| ≤40 000 | 8 317 (41.66) | 6 264 (39.85) | <0.001 |
| 40 000-99 999 | 8 902 (44.59) | 7 133 (45.38) |  |
| ≥100 000 | 2 640 (13.22) | 2 231 (14.19) |  |
| Total physical activity, MET-h/day | 11.20 (15.30) | 11.85 (15.93) | <0.001 |
| Diet |  |  |  |
| Fruit, n (%), times/ week |  |  |  |
| Never | 7 304 (36.58) | 5 651 (35.95) | 0.763 |
| 1-3 | 6 316 (31.63) | 5 087 (32.36) |  |
| 4-6 | 741 ( 3.71) | 580 ( 3.69) |  |
| ≥7 | 2 786 (13.95) | 2 174 (13.83) |  |
| Less than weekly | 2 301 (11.52) | 1 812 (11.53) |  |
| Vegetable, n (%), times/ week |  |  |  |
| Never | 193 ( 0.97) | 148 ( 0.94) | 0.976 |
| 1-3 | 360 ( 1.80) | 280 ( 1.78) |  |
| 4-6 | 244 ( 1.22) | 177 ( 1.13) |  |
| ≥7 | 18 247 (91.39) | 14 380 (91.49) |  |
| Less than weekly | 32 ( 0.16) | 25 ( 0.16) |  |
| Red meat, n (%), times/ week |  |  |  |
| Never | 1 302 ( 6.52) | 919 ( 5.85) | 0.025 |
| 1-3 | 9 878 (49.47) | 7 856 (49.98) |  |
| 4-6 | 1 943 ( 9.73) | 1 566 ( 9.96) |  |
| ≥7 | 4 603 (23.05) | 3 713 (23.62) |  |
| Less than weekly | 1 639 ( 8.21) | 1 193 ( 7.59) |  |
| *Abbreviation: BMI, body mass index; MET, metabolic equivalent tasks For some variables, the totals did not sum to 100% due to small proportions of participants choosing "prefer not to answer". | | | |

| Supplement Table 3. Association between tea consumption and all-cause mortality risk stratified by potential risk factors | | | | |
| --- | --- | --- | --- | --- |
| Characteristics | Frequency of tea consumption (times/week) | | | *P* for trend |
|  | Never | 1-2 | ≥3 |  |
| Excluding deaths within the first 2 years of follow-up （n=348） | | | | |
| Death /Total person year | 1 879 / 95 627 | 164 / 9 999 | 658 / 35 349 |  |
| Model 1 | 1.00 | 0.77 ( 0.66 , 0.91 ) | 0.77 ( 0.69 , 0.86 ) | <0.001 |
| Model 2 | 1.00 | 0.81 ( 0.69 , 0.96 ) | 0.83 ( 0.74 , 0.92 ) | <0.001 |
| Excluding diabetes duration ≤ 1 year（n=3 479） | | | | |
| Death /Total person year | 1 700 / 72 978 | 152 / 7 945 | 610 / 28 824 |  |
| Model 1 | 1.00 | 0.72 ( 0.61 , 0.86 ) | 0.71 ( 0.64 , 0.79 ) | <0.001 |
| Model 2 | 1.00 | 0.77 ( 0.65 , 0.91 ) | 0.78 ( 0.70 , 0.88 ) | <0.001 |
| Excluding accidental deaths（n=193） | | | | |
| Death /Total person year | 1 994 / 95 223 | 171 / 9 934 | 691 / 35 181 |  |
| Model 1 | 1.00 | 0.75 ( 0.64 , 0.88 ) | 0.75 ( 0.68 , 0.83 ) | <0.001 |
| Model 2 | 1.00 | 0.79 ( 0.67 , 0.93 ) | 0.81 ( 0.73 , 0.90 ) | <0.001 |
| Excluding coffee consumption（n=187） | | | | |
| Death /Total person year | 2 117 / 95 145 | 184 / 9 824 | 731 / 34 675 |  |
| Model 1 | 1.00 | 0.76 ( 0.65 , 0.89 ) | 0.75 ( 0.68 , 0.83 ) | <0.001 |
| Model 2 | 1.00 | 0.81 ( 0.69 , 0.94 ) | 0.81 ( 0.73 , 0.90 ) | <0.001 |
| HRs were calculated in Cox proportional hazards model. Model1 was adjusted for age (years), sex (male, female). Model 2 was further adjusted for educational level (without formal education, primary and middle school, high school or above, unknown), marital status(married, unmarried),annual household income (< 40 000, 40 000-99 999, ≥ 100 000 yuan, unknown), smoking status (never, previous, current, unknown), alcohol drinking status (never, previous, current, unknown), body mass index (kg/m^2^), total physical activity (MET-h/day), duration of diabetes (years), oral antidiabetic medication use (no, yes), insulin use (no, yes), fruit consumption (never, 1-3 times per week, 4-6 times per week, ≥ 7 times per week, less than weekly), vegetable consumption (never, 1-3 times per week, 4-6 times per week, ≥ 7 times per week, less than weekly), animal meat consumption (never, 1-3 times per week, 4-6 times per week, ≥ 7 times per week, less than weekly). | | | | |

| Supplement Table 4. HR (95% CI) of all-cause and cause-specific mortality according to tea consumption characteristics(n = 19 966) | | | | | | | | | |
| --- | --- | --- | --- | --- | --- | --- | --- | --- | --- |
| Characteristics | All mortality（n=4 452） | | | CVD mortality（n=1 522） | | | cancer mortality（n=1 069） | | |
|  | Death /Total person year | *HR* (95%*CI*) | | Death /Total person year | *HR* (95%*CI*) | | Death /Total person year | *HR* (95%*CI*) | |
|  |  | Model 1 | Model 2 |  | Model 1 | Model 2 |  | Model 1 | Model 2 |
| Frequency of tea consumption (times/week) | | | | | | | | | |
| Never | 3 106 / 121 089 | 1.00 | 1.00 | 1 099 / 121 089 | 1.00 | 1.00 | 685 / 121 089 | 1.00 | 1.00 |
| 1-2 | 265 / 12 365 | 0.77 ( 0.67 , 0.87 ) | 0.81 ( 0.71 , 0.92 ) | 82 / 12 365 | 0.69 ( 0.55 , 0.86 ) | 0.72 ( 0.57 , 0.91 ) | 70 / 12 365 | 0.84 ( 0.66 , 1.09 ) | 0.90 ( 0.69 , 1.16 ) |
| ≥3 | 1 081 / 43 481 | 0.78 ( 0.72 , 0.84 ) | 0.84 ( 0.77 , 0.91 ) | 371 / 43 481 | 0.78 ( 0.68 , 0.90 ) | 0.86 ( 0.75 , 1.00 ) | 314 / 43 481 | 0.91 ( 0.77 , 1.06 ) | 0.97 ( 0.83 , 1.14 ) |
| *P* for trend |  | <0.001 | <0.001 |  | <0.001 | 0.030 |  | 0.193 | 0.720 |
| Consumption grams of tea per day(grams/day) | | | | | | | | | |
| Never | 3 106 / 121 089 | 1.00 | 1.00 | 1 099 / 121 089 | 1.00 | 1.00 | 685 / 121 089 | 1.00 | 1.00 |
| 1-4 | 661 / 26 537 | 0.80 ( 0.73 , 0.88 ) | 0.86 ( 0.78 , 0.94 ) | 237 / 26 537 | 0.82 ( 0.70 , 0.95 ) | 0.89 ( 0.76 , 1.04 ) | 171 / 26 537 | 0.86 ( 0.72 , 1.04 ) | 0.92 ( 0.77 , 1.11 ) |
| >4 | 523 / 22 224 | 0.73 ( 0.66 , 0.81 ) | 0.79 ( 0.71 , 0.88 ) | 153 / 22 224 | 0.62 ( 0.52 , 0.75 ) | 0.69 ( 0.57 , 0.83 ) | 167 / 22 224 | 0.93 ( 0.77 , 1.13 ) | 1.02 ( 0.83 , 1.24 ) |
| *P* for trend |  | <0.001 | <0.001 |  | <0.001 | <0.001 |  | 0.330 | 0.984 |
| Consumption cups of tea per day(cups/day) | | | | | | | | | |
| Never | 3 106 / 121 089 | 1.00 | 1.00 | 1 099 / 121 089 | 1.00 | 1.00 | 685 / 121 089 | 1.00 | 1.00 |
| 1 | 901 / 36 480 | 0.75 ( 0.69 , 0.82 ) | 0.81 ( 0.74 , 0.88 ) | 306 / 36 480 | 0.74 ( 0.64 , 0.86 ) | 0.81 ( 0.70 , 0.94 ) | 243 / 36 480 | 0.84 ( 0.71 , 0.99 ) | 0.91 ( 0.77 , 1.08 ) |
| ≥2 | 403 / 17 603 | 0.81 ( 0.73 , 0.91 ) | 0.87 ( 0.78 , 0.98 ) | 130 / 17 603 | 0.78 ( 0.64 , 0.95 ) | 0.84 ( 0.69 , 1.03 ) | 129 / 17 603 | 1.01 ( 0.82 , 1.24 ) | 1.06 ( 0.86 , 1.31 ) |
| *P* for trend |  | <0.001 | <0.001 |  | <0.001 | 0.015 |  | 0.530 | 0.890 |
| Duration of tea consumption (years) | | | | | | | | | |
| Never | 3 106 / 121 089 | 1.00 | 1.00 | 1 099 / 121 089 | 1.00 | 1.00 | 685 / 121 089 | 1.00 | 1.00 |
| 1-30 | 378 / 23 590 | 0.79 ( 0.71 , 0.88 ) | 0.82 ( 0.74 , 0.92 ) | 123 / 23 590 | 0.77 ( 0.64 , 0.94 ) | 0.80 ( 0.66 , 0.98 ) | 105 / 23 590 | 0.84 ( 0.67 , 1.04 ) | 0.87 ( 0.70 , 1.09 ) |
| >30 | 759 / 24 986 | 0.72 ( 0.66 , 0.79 ) | 0.78 ( 0.71 , 0.86 ) | 253 / 24 986 | 0.69 ( 0.59 , 0.81 ) | 0.76 ( 0.65 , 0.90 ) | 217 / 24 986 | 0.86 ( 0.72 , 1.02 ) | 0.93 ( 0.77 , 1.12 ) |
| *P* for trend |  | <0.001 | <0.001 |  | <0.001 | <0.001 |  | 0.062 | 0.381 |
| HRs were calculated in Cox proportional hazards model. Model1 was adjusted for age (years), sex (male, female). Model 2 was further adjusted for educational level (without formal education, primary and middle school, high school or above, unknown), marital status(married, unmarried),annual household income (< 40 000, 40 000-99 999, ≥ 100 000 yuan, unknown), smoking status (never, previous, current, unknown), alcohol drinking status (never, previous, current, unknown), body mass index (kg/m2), total physical activity (MET-h/day), duration of diabetes (years), oral antidiabetic medication use (no, yes), insulin use (no, yes),fruit consumption (never, 1-3 times per week, 4-6 times per week, ≥ 7 times per week, less than weekly), vegetable consumption (never, 1-3 times per week, 4-6 times per week, ≥ 7 times per week, less than weekly), animal meat consumption (never, 1-3 times per week, 4-6 times per week, ≥ 7 times per week, less than weekly). | | | | | | | | | |

| Supplement Table 5. HR (95% CI) of all-cause and cause-specific mortality according to tea consumption characteristics(n = 15 448) | | | | | | | | | |
| --- | --- | --- | --- | --- | --- | --- | --- | --- | --- |
| Characteristics | All-cause mortality（n=3 008） | | | CVD mortality（n=907） | | | cancer mortality（n=725） | | |
|  | Death /Total person year | *HR* (95%*CI*) | | Death /Total person year | *HR* (95%*CI*) | | Death /Total person year | *HR* (95%*CI*) | |
|  |  | Model 1 | Model 2 |  | Model 1 | Model 2 |  | Model 1 | Model 2 |
| Frequency of tea consumption (times/week) | | | | | | | | | |
| Never | 2 123 / 95 914 | 1.00 | 1.00 | 654 / 95 914 | 1.00 | 1.00 | 461 / 95 914 | 1.00 | 1.00 |
| 1-2 | 176 / 9 290 | 0.79 ( 0.67 , 0.92 ) | 0.83 ( 0.71 , 0.98 ) | 50 / 9 290 | 0.75 ( 0.56 , 1.01 ) | 0.79 ( 0.58 , 1.06 ) | 46 / 9 290 | 0.87 ( 0.64 , 1.19 ) | 0.91 ( 0.67 , 1.25 ) |
| ≥3 | 709 / 33 685 | 0.76 ( 0.68 , 0.84 ) | 0.82 ( 0.74 , 0.91 ) | 203 / 33 685 | 0.73 ( 0.61 , 0.88 ) | 0.79 ( 0.65 , 0.96 ) | 218 / 33 685 | 0.96 ( 0.79 , 1.16 ) | 1.03 ( 0.84 , 1.26 ) |
| *P* for trend |  | <0.001 | <0.001 |  | <0.001 | 0.015 |  | 0.641 | 0.779 |
| Consumption grams of tea per day(grams/day) | | | | | | | | | |
| Never | 2 123 / 95 914 | 1.00 | 1.00 | 654 / 95 914 | 1.00 | 1.00 | 461 / 95 914 | 1.00 | 1.00 |
| 1-4 | 431 / 20 419 | 0.78 ( 0.70 , 0.87 ) | 0.83 ( 0.74 , 0.94 ) | 133 / 20 419 | 0.79 ( 0.64 , 0.97 ) | 0.84 ( 0.68 , 1.03 ) | 114 / 20 419 | 0.89 ( 0.71 , 1.11 ) | 0.94 ( 0.75 , 1.19 ) |
| >4 | 351 / 17 770 | 0.71 ( 0.63 , 0.81 ) | 0.77 ( 0.68 , 0.88 ) | 85 / 17 770 | 0.58 ( 0.45 , 0.75 ) | 0.63 ( 0.48 , 0.81 ) | 121 / 17 770 | 1.01 ( 0.80 , 1.27 ) | 1.10 ( 0.87 , 1.41 ) |
| *P* for trend |  | <0.001 | <0.001 |  | <0.001 | <0.001 |  | 0.916 | 0.507 |
| Consumption cups of tea per day(cups/day) | | | | | | | | | |
| Never | 2 123 / 95 914 | 1.00 | 1.00 | 654 / 95 914 | 1.00 | 1.00 | 461 / 95 914 | 1.00 | 1.00 |
| 1 | 585 / 27 770 | 0.73 ( 0.66 , 0.81 ) | 0.80 ( 0.71 , 0.89 ) | 161 / 27 770 | 0.67 ( 0.55 , 0.82 ) | 0.72 ( 0.59 , 0.88 ) | 168 / 27 770 | 0.90 ( 0.73 , 1.10 ) | 0.97 ( 0.79 , 1.20 ) |
| ≥2 | 270 / 13 856 | 0.82 ( 0.71 , 0.94 ) | 0.86 ( 0.75 , 0.99 ) | 80 / 13 856 | 0.84 ( 0.65 , 1.08 ) | 0.88 ( 0.68 , 1.14 ) | 89 / 13 856 | 1.05 ( 0.82 , 1.36 ) | 1.10 ( 0.85 , 1.42 ) |
| *P* for trend |  | <0.001 | 0.002 |  | 0.001 | 0.057 |  | 0.977 | 0.593 |
| Duration of tea consumption (years) | | | | | | | | | |
| Never | 2 123 / 95 914 | 1.00 | 1.00 | 654 / 95 914 | 1.00 | 1.00 | 461 / 95 914 | 1.00 | 1.00 |
| 1-30 | 237 / 17954 | 0.80 ( 0.69 , 0.92 ) | 0.83 ( 0.72 , 0.96 ) | 60 / 17 954 | 0.72 ( 0.54 , 0.95 ) | 0.74 ( 0.56 , 0.97 ) | 69 / 17 954 | 0.87 ( 0.66 , 1.14 ) | 0.90 ( 0.68 , 1.18 ) |
| >30 | 512 / 19731 | 0.70 ( 0.63 , 0.78 ) | 0.77 ( 0.68 , 0.86 ) | 151 / 19 731 | 0.69 ( 0.56 , 0.85 ) | 0.75 ( 0.6 , 0.93 ) | 154 / 19 731 | 0.91 ( 0.74 , 1.13 ) | 1.00 ( 0.8 , 1.26 ) |
| *P* for trend |  | <0.001 | <0.001 |  | <0.001 | 0.005 |  | 0.372 | 0.96 |
| Type of tea | | | | | | | | | |
| Never | 2 123 / 95 914 | 1.00 | 1.00 | 654 / 95 914 | 1.00 | 1.00 | 461 / 95 914 | 1.00 | 1.00 |
| Green tea | 856 / 41 121 | 0.76 ( 0.70 , 0.84 ) | 0.82 ( 0.75 , 0.91 ) | 239 / 41 121 | 0.72 ( 0.60 , 0.86 ) | 0.77 ( 0.64 , 0.92 ) | 258 / 41 121 | 0.95 ( 0.79 , 1.15 ) | 1.02 ( 0.84 , 1.24 ) |
| Black tea | 13 / 780 | 0.78 ( 0.45 , 1.34 ) | 0.92 ( 0.53 , 1.59 ) | 6 / 780 | 1.24 ( 0.55 , 2.79 ) | 1.39 ( 0.61 , 3.17 ) | 3 / 780 | 0.70 ( 0.23 , 2.20 ) | 0.77 ( 0.25 , 2.41 ) |
| Oolong tea | 12 / 624 | 0.92 ( 0.52 , 1.63 ) | 0.90 ( 0.51 , 1.60 ) | 7 / 624 | 1.92 ( 0.91 , 4.08 ) | 1.81 ( 0.85 , 3.87 ) | 3 / 624 | 0.87 ( 0.28 , 2.74 ) | 0.83 ( 0.27 , 2.62 ) |
| *P* for trend |  | - | - |  | - | - |  | - | - |
| HRs were calculated in Cox proportional hazards model. Model1 was adjusted for age (years), sex (male, female). Model 2 was further adjusted for educational level (without formal education, primary and middle school, high school or above, unknown), marital status(married, unmarried),annual household income (< 40 000, 40 000-99 999, ≥ 100 000 yuan, unknown), smoking status (never, previous, current, unknown), alcohol drinking status (never, previous, current, unknown), body mass index (kg/m^2^), total physical activity (MET- h/day), duration of diabetes (years), oral antidiabetic medication use (no, yes), insulin use (no, yes),fruit consumption (never, 1-3 times per week, 4-6 times per week, ≥ 7 times per week, less than weekly), vegetable consumption (never, 1-3 times per week, 4-6 times per week, ≥ 7 times per week, less than weekly), animal meat consumption (never, 1-3 times per week, 4-6 times per week, ≥ 7 times per week, less than weekly). | | | | | | | | | |
