# Supplementary material for: Associations of Tea Consumption With the Risk of All‐Cause and Cause‐Specific Mortality Among Adults With Type 2 Diabetes: A Prospective Cohort Study in China
Source: J Diabetes. 2025 Jan 20;17(1):e70040. doi: 10.1111/1753-0407.70040 (PMC11744464; doi:10.1111/1753-0407.70040)
Supplement: Supplementary file 1 — Data S1. [file JDB-17-e70040-s001.zip › K201105.pdf]

科 技 项 目 课 题 合 同

课题编号：K201105

课题名称：基于社区的大规模人群2型糖尿病综合干预及应用研究

承担单位：江苏省公共卫生研究院

课题负责人：武鸣

起止年限：2011.1-2013.12

江 苏 省 卫 生 厅 制

二零一一 年 十一 月 三十 日

一、 研究内容和目标（包括阶段性目标、最终目标）及主要技术经济指标。

1.研究目标

鉴于2型糖尿病（T2DM）对我国居民健康的重大危害和长期持续的巨大负担，本研究拟在国家“十一五”科技支撑计划“代谢综合征的发病趋势和综合控制研究”（2006BAI01A01）和卫生部重点课题 “江苏省多代谢异常与代谢综合征综合防治研究” （WKJ2004-2-014）等多项科研基础上,在我省进行以社区为基础的大规模糖尿病综合干预研究：即通过连续动态收集糖尿病患者环境、营养、生活方式等流行病学资料和空腹血糖、糖化血红蛋白、血脂等临床关键指标，客观评价糖尿病患者血糖控制效果，研究糖尿病并发症及其环境、行为和遗传影响因素，继而有针对性地对血糖控制未达标对象进行基于信息化平台的个体化健康教育，营养、膳食多样化指导，以及在运动与生活方式基础上的遗传敏感药物优化综合干预；研究结果将显著改善研究社区人群糖尿病控制效果，降低或延缓并发症的发生与发展，并形成可推广应用的社区糖尿病强化干预适宜技术。此外还将筛选影响人群糖尿病疗效、并发症及其预后的遗传易感基因与信号调控miRNA，为进一步设计敏感分子药物提供理论依据，这将有助于阐明影响我国人群抗糖尿病药物疗效及预后的分子与遗传机制，为糖尿病患者药物敏感性和预后预测提供客观指标，以及为进一步设计敏感分子药物提供理论依据。

2. 主要研究内容

目前本团队已在省内对2万余名T2DM患者进行了2年的人群随访观察，登记了患者用药的种类、剂量并形成初步的疗效判断，连续动态收集了患者空腹血糖、人口学资料及吸烟、饮酒、膳食等主要行为危险因素信息。本研究将进一步连续动态收集患者糖化血红蛋白、血脂等血糖控制关键指标，系统评价血糖控制效果，糖尿病并发症的发生情况及其环境、行为和遗传影响因素，并展开敏感指标筛选和综合预测分析，应用危险因素评分方法建立疗效与预后的预测预警模型。具体研究内容包括：

（1）分析糖尿病疗效及并发症的影响因素，建立社区预测预警模型

对前期已纳入管理2年的2万余名T2DM患者人群随访观察记录进行分类整理，登记用药的种类、剂量，开展流行病学调查并收集相关个体信息及生活方式特征，同时检测患者糖化血红蛋白、胰岛素、血脂等血糖控制关键指标，对使用药物治疗人的群进行疗效评价，系统评价血糖控制效果；分析糖尿病并发症及其环境、行为和遗传影响因素；并在此基础上开展敏感指标筛选和综合预测分析，建立糖尿病疗效与预后的预测预警模型。

（2）设计优化的综合干预方案，探讨建立可推广的社区糖尿病综合防治模式

应用健康教育和健康促进手段，利用网络在我省“居民健康素养学习系统”中开辟糖尿病相关知识学习和评估窗口，同时通过有线电视、网络、手机短信提醒等新模式为患者提供全方位和个体化服务，实现公众健康教育互动。对血糖难以控制的对象进行基于信息化平台的强化健康教育，根据患者个体的实际需求和管理需要，开展营养和膳食干预，制定个体化的膳食多样化指南，设计食物种类登记表和记录提醒程序；根据全民健身运动指南，开展且为大多数患病对象接受的娱乐型体力活动方案，设计个体化运动安排和时间长度、频度以及记录、提醒程序；设计针对超重/肥胖、血脂异常、血压升高等高危人群的个体化生活方式干预方案等。

在膳食多样化、运动与生活方式基础上，还将针对遗传与行为生活方式特点对患者进行药物敏感性分析，选择适合不同类型患者的经济型单一用药或联合治疗方案，最终形成可推广的社区糖尿病药物和非药物干预适宜技术，探索社区糖尿病综合防治模式。

（3）药物基因组学研究

通过不同疗效与并发症发生的人群比较研究，筛选影响人群糖尿病疗效与并发症及其预后的遗传易感基因及其信号调控 miRNA。具体内容包括：选择用药治疗 6 个月以上 HbA1c≥7%的 T2DM 作为病例组，选择用药 6 个月以上 HbA1c<7%的患者作为对照组，按年龄和性别进行匹配。根据已经完成的生物信息学分析以及获得的中国人群数据验证，与 T2DM 关联的基因位点（SNPs）共有 58 个，另选择其中 16 个功能明确基因的 miRNA 候选 SNPs 42 个，共纳入 100 个 SNPs 进行关联分析。应用 TaqMan 探针技术进行 SNP 基因分型，通过比较不同血糖控制效果人群候选基因频率差异的关联分析筛选易感 SNPs，并采用目前国际上广泛应用的 GMDR 方法构建非参数回归模型，分析基因-基因与基因-环境交互作用，及应用遗传危险评分的方法建立药物疗效及预后预测模型。

3. 预期成果

（1）建立社区糖尿病患者疗效与预后的预测预警模型。

（2）开发多项社区糖尿病综合干预适宜技术，如网络学习和评估系统，糖尿病患者健康教育系列短片，糖尿病患者健康管理系统等。

（3）筛选影响人群糖尿病疗效与心血管并发症及其预后的 SNPs 5-8 个，申报有关药物敏感性研究专利 1-2 项。

（4）社区干预人群糖尿病患者血糖控制率比现有基础提高 50%以上。

（5）发表论文 10-15 篇，其中 SCI 论文 4-6 篇，培养研究生 4-5 名。

计划进度（总的研究期限，进度及分年度计划）

2011 年

（1）进行糖尿病患者随访，覆盖 2 万人群，开展流行病学调查；

（2）收集符合条件病人的生物样本，检测糖化血红蛋白，相关实验条件准备；

（3）建立以互联网为载体的信息平台；

（4）进行综合干预方案设计，开展干预预实验；

（5）开展糖尿病患者人群干预；

（6）召开一次糖尿病预防控制研讨会，组织省内学术交流 1 次；

（7）发表学术论文 2 篇；

2012 年

（1）进行第二年度糖尿病患者随访；

（2）收集符合条件病人的生物样本，检测糖化血红蛋白，提取 DNA；

（3）开展糖尿病疗效及并发症的影响因素分析；

（4）开展糖尿病治疗的药物基因组学研究，筛选影响糖尿病疗效与并发症 SNPs3-4 个；

（5）初步建立糖尿病药物疗效及预后预测预警模型；

（6）继续开展糖尿病人群干预，开发或引进一项干预适宜技术；

（7）参加国内糖尿病学术交流 1 次；

（8）发表论文 5 篇；包括 SCI 文章 1 篇

（9）培养研究生 1 名；

2013 年

（1）进行第三年度糖尿病患者随访；

（2）收集符合条件病人的生物样本，检测糖化血红蛋白；

（3）继续糖尿病药物基因组学研究，筛选影响糖尿病疗效与并发症 SNPs3-4 个；

（4）修正糖尿病药物疗效及预后预警预测模型；

（5）继续开展糖尿病人群干预，干预人群血糖控制率比基线基础提高 50%以上；

（6）组织或参加国际学术交流 1 次；

（7）发表论文 5 篇，包括 SCI 文章 2 篇

（8）培养研究生 1 名；

（9）推广社区糖尿病适宜技术和干预模式；

（10）总结药物基因组学研究结果，申报有关药物敏感性研究专利 1-2 项；

（11）数据整理，课题总结；

课题人员情况（共计最多填报 15 人）

姓　名	性别	出生年月	职务/职称	业务专业	为本课题工作时间(%)	所在单位
课题负责人						
武鸣	男	1974 年 7 月	副主任医师	流行病学	40	江苏省公共卫生研究院慢性疾病防制研究所
主要研究人员						
黄明豪	男	1953.9	研究员	健康教育	40	江苏省公共卫生研究院慢性疾病防制研究所
甄世祺	男	1974.3	副主任医师	流行病学	40	江苏省公共卫生研究院慢性疾病防制研究所
沈冲	男	1973.1	副教授	流行病学	40	江苏省公共卫生研究院慢性疾病防制研究所
张永青	女	1969.4	副主任医师	流行病学	50	江苏省公共卫生研究院慢性疾病防制研究所
李小宁	男	1963.4	主任医师	流行病学	40	江苏省公共卫生研究院慢性疾病防制研究所
史祖民	男	1964.12	副主任医师	流行病学	30	江苏省公共卫生研究院慢性疾病防制研究所
陶然	男	1977.10	副主任医师	流行病学	50	江苏省公共卫生研究院慢性疾病防制研究所
左辉	男	1979.11	主管医师	流行病学	50	江苏省公共卫生研究院慢性疾病防制研究所
胡晓抒	男	1956.10	主任医师	公共卫生	20	江苏省公共卫生研究院慢性疾病防制研究所

二、 签约各方共同商定的条款

1. 经费核定数

经费来源	年度经费				
	2011 年	2012 年	2013 年	200 年	200 年
总经费	7	7	6		
省卫生厅拨款	3	4	3		
其它部门拨款					
承担单位自筹	4	3	3		
外汇					

注：单位自筹及外汇请注明来源

2. 拨款偿还计划

课题类别	偿还金额	偿还计划				
		200 年	200 年	200 年	200 年	200 年

3. 依附应按期偿还甲方经费，乙方如遇特殊情况无法按期还款是，必须在规定还款期之前向甲方提出书面报告，经甲方正式行文同意后才能准予延期还款，同时确定还款日期。
4. 甲方同意乙方购买以下设备：
5. 在合同执行过程中，任何一方不得擅自修改合同内容，如确需要修改，应按计划管理办法规定修改。
6. 甲方中途无故撤销或不履行合同时，所拨经费、物资不得追回，乙方如无正常理由不履行合同，或非不可抗拒的客观原因，致使合同无法执行时，应全部退回所拨经费物资。
7. 丙方应监督检查并保证合同条款执行，协助解决合同执行过程中出现的问题，积极解决合同中应属本部门解决的问题。
8. 甲、乙、丙各方对技术资料有保密责任。
9. 签约各方都必须执行《江苏省卫生厅科研项目管理细则》第十章“课题研究过程管理”的各项条款。
10. 本合同一式五份，分存甲方二份、乙方二份、丙方一份。
11. 本合同协议的其他条款如下：

签订合同各方：

委托单位（甲方）：

负责人（签字）　　　　　　　　　　　　　　　　公　　章

经办人（签字）

2011 年 11 月 30 日

开户银行、帐号：

承担单位（乙方）：江苏省公共卫生研究院（江苏省疾病预防控制中心）

负责人（签字）

课题负责人（签字）　　　　　　　　　　　　　　公　　章

财务部门负责人（签字）　　　　　　　　　　　　2011 年 11 月 3 日

开户银行、帐号：　　南京民生银行北京西路支行　0802 0142 1000 2340

江苏省疾病预防控制中心
08020142100023[illegible]
南京民生银行北京西路支行

保证单位（丙方）：　　　　　　　　　　　　　　公　　章

负责人（签字）　　　　　　　　　　　　　　　　年　　月　　日
