# Supplementary material for: Associations of Tea Consumption With the Risk of All‐Cause and Cause‐Specific Mortality Among Adults With Type 2 Diabetes: A Prospective Cohort Study in China
Source: J Diabetes. 2025 Jan 20;17(1):e70040. doi: 10.1111/1753-0407.70040 (PMC11744464; doi:10.1111/1753-0407.70040)
Supplement: Supplementary file 1 — Data S1. [file JDB-17-e70040-s001.zip › M2020085.pdf]

关于公布江苏省卫生健康委2020年度医学科研立项项目的通知（苏卫科教〔2020〕11号）

发布日期：2020-11-17 11:17:49 浏览次数：2381

各设区市卫生健康委，昆山、泰兴、沭阳县（市）卫生健康委，各有关单位：

根据《关于组织开展省卫生健康委2020年度医学科研项目申报工作的通知》（苏卫科教〔2020〕7号）精神，按照省卫生健康委科研管理的程序和要求，经各单位遴选推荐，我委组织开展了2020年度医学科研项目评审工作。根据评审和公示结果，确定苏州大学附属第一医院陈亮的“多相微纳米仿生骨膜促进骨缺损修复的研究”等63个研究项目为重点项目，江苏省中医院赵苏苏的“基于CD33介导的砒霜纳米粒靶向急性早幼粒细胞白血病递药系统的构建与评价”等105个项目为面上项目，滨海县人民医院钱雷的“IL-35调控NLRP3/caspase-1信号通路降低缺血再灌注诱导的血脑屏障通透性的机制研究”等80个项目为指导性项目（各类项目立项情况见附件）。

希望各单位高度重视医学科研工作，按规定落实项目配套经费，严格规范经费管理；认真完成研究任务，确保研究质量。同时，积极推动科研成果应用转化，为增强疾病防治能力和水平、推动卫生健康事业健康发展提供科技支撑。

本次评审确认的所有项目，均需与我委签订《江苏省卫生健康委科研项目合同书》。合同书签订须登录“江苏省卫生健康委科研管理平台”（https://58.213.112.246/wskj/）在线填报（网络开放时间为2020年11月16日9:00-11月29日17:00），经我委审核通过后，打印含水印的正式合同书。纸质合同书一式四份于12月6日前寄送我委科教处（地址：南京市中央路42号）。

联系人：叶荣、李艳，电话：025-83620705、83620703。

附件：省卫生健康委2020年度医学科研立项项目

江苏省卫生健康委员会

2020年11月9日

附件下载：附件：省卫生健康委2020年度医学科研立项项目.pdf

附件

省卫生健康委 2020 年度医学科研立项项目

序号	项目名称	项目负责人	申报单位	立项类型
1	多相微纳米仿生骨膜促进骨缺损修复的研究	陈　亮	苏州大学附属第一医院	重点 A 类项目
2	合欢皮通过催产素调控前额叶–边缘系统神经环路对勃起功能调节机制研究	陈　赟	江苏省中医院	重点 A 类项目
3	基于精准治疗的 H 型高血压人群卒中的二级预防的研究	李爱民	连云港市第一人民医院	重点 A 类项目
4	GSK3 调节 ENO1 在 MLL 基因重排白血病中作用机制的研究	李天宇	无锡市儿童医院	重点 A 类项目
5	凋亡特异性 PET 分子探针的构建及其在肿瘤放化疗早期疗效评价中的应用研究	林建国	江苏省原子医学研究所	重点 A 类项目
6	大肠杆菌噬菌体 EG1 裂解酶功能改造与应用	刘根焰	江苏省人民医院	重点 A 类项目
7	基于荧光免疫技术的献血者初筛快速检测技术研究	栾建凤	东部战区总医院	重点 A 类项目
8	微纳流体传感器快速检测外泌体 lncRNA 在卵巢癌早期筛查中的应用	沈　杨	东南大学附属中大医院	重点 A 类项目
9	基于介孔碳的靶向复合纳米系统递送 circ_0064555 抑制胶质瘤恶性生长的作用及机制研究	施金龙	南通大学附属医院	重点 A 类项目

序号	项目名称	项目负责人	申报单位	立项类型
106	SIRT1 对多巴胺能神经元及帕金森模型的影响的研究	罗　镧	南通大学附属医院	面上项目
107	基于“护联网”的医院–社区老年健康照护模式构建及应用研究	莫永珍	江苏省省级机关医院	面上项目
108	放疗中超声图像的人工智能自动勾画技术及其临床应用研究	倪昕晔	常州市第二人民医院	面上项目
109	肿瘤生物样本库资源共享模式及评价体系研究	潘　睿	江苏省肿瘤医院	面上项目
110	肿瘤外泌体来源的 miR–1290 靶向抑制 PPARα 分子驯化肿瘤相关成纤维细胞促进肺癌进展的作用机制研究	潘金顺	南京医科大学第二附属医院	面上项目
111	基于 3D–PCX 耐药模型的胶质母细胞瘤逆转抗药策略	浦浙宁	无锡市人民医院	面上项目
112	RNA 去甲基酶 FTO 缺失通过下调 SOX17 致精子发生障碍机制研究	钱春凤	苏州市立医院	面上项目
113	乙型肝炎患者 HBV 特异性 T 细胞免疫数据库的建立及临床应用研究	邱　洁	南京市第二医院	面上项目
114	FSP1 在儿童紫癜性肾炎足细胞损伤中的作用和机制	任献国	南京医科大学附属逸夫医院	面上项目
115	基于 B 型主动脉夹层形态和血流功能参数的智能诊断关键技术研究	邵　峻	南京医科大学第二附属医院	面上项目
116	2 型糖尿病患者长期血糖变异性与心血管病发生和死亡风险的前瞻性研究	苏　健	江苏省疾病预防控制中心	面上项目
