# Supplementary material for: Associations of Tea Consumption With the Risk of All‐Cause and Cause‐Specific Mortality Among Adults With Type 2 Diabetes: A Prospective Cohort Study in China
Source: J Diabetes. 2025 Jan 20;17(1):e70040. doi: 10.1111/1753-0407.70040 (PMC11744464; doi:10.1111/1753-0407.70040)
Supplement: Supplementary file 1 — Data S1. [file JDB-17-e70040-s001.zip › SS202010.pdf]

计划类别：民生科技->科技示范工程

苏 州 市 科 技 计 划 项 目 合 同

项目编号 SS202010

项目名称 基于人工智能和大数据的糖尿病综合防控体系构建与科技示范

起止年限 2020 年 07 月 01 日至 2023 年 06 月 30 日

承担单位 苏州市疾病预防控制中心

单位地址 苏州市三香路 72 号

邮　　编 215004

项目负责人 陆艳 电话 18962168795

财务负责人 周坚钢 电话 0512-68262517

项目联系人 陆艳 电话 18962168795

苏州市科学技术局

二〇一九年制

基于人工智能和大数据的糖尿病综合防控体系构建与科技示范　项目经苏财教〔2020〕115号文批准列入苏州市2020年科技发展计划正式下达。为加强科技计划项目的实施与管理，根据中华人民共和国合同法规定，甲方——苏州市科学技术局，乙方—— 苏州市疾病预防控制中心，丙方——苏州市卫生健康委员会，就有关事宜签订本科技计划项目合同。

一、项目的目标和主要研究内容

要解决的主要技术难题和问题，项目研究的创新点和内容：

要解决的主要技术难题和问题：

1. 人工智能化的糖尿病高危人群筛查及诊断。2. 人机对话实现随访干预。3. 运用AI糖尿病知识图谱进行饮食记录及针对性的指导建议。4. 通过佩戴穿戴设备来记录运动的方式和时长。5. 建立AI糖尿病及其并发症的数据预测模型。6. 和医疗大数据互联互通，构建家庭-社区-疾控-医院的四维管理模式。7. 建立苏州市糖尿病大型队列，进行跟踪随访。8. 利用人工智能，及时提醒居民及家属关注糖尿病。9. 若糖尿病患者出现异常，人工智能化提醒其家庭医生，及时进行诊治。10. 健全糖尿病综合防治服务体系，推进糖尿病防、治、管整体融合发展。

项目研究的创新点和内容：

运用AI技术与大数据对糖尿病进行同质化社区防控，建立糖尿病及其并发症预测模型指导糖尿病病人实现个性化精准治疗。

关键技术：

人工智能糖尿病综合管理平台构建家庭-社区-疾控-医院的四维管理模式。

二、项目验收内容和考核指标

1、　项目预期成果类型和数量

成果形式（可多选）	☐1. 专利
	☑2. 论文论著
	☐3. 技术标准
	☐4. 新产品（含农业新产品）
	☐5. 新装备（装置）
	☐6. 新工艺（或新方法、新模式）
	☐7. 新材料

	☑8. 计算机软件
	☑9. 研究(咨询)报告
	☐10. 基地建设
	☐11. 新的服务模式
	☐12. 其它

专利申请（件）	发明	0
	实用新型	0
	外观设计	0
专利授权（件）	发明	0
	实用新型	0
	外观设计	0
发表论文（篇）	论文总数	4
	其中核心期刊	2
	其中科学引文索引（SCI）	1
	其中工程索引（EI）	0
出版科技著作（部）		0
制定技术标准（个）		0
其中：国际、国家标准（个）		0
新 产 品（个）		0
农业新产品（个）		0
新装备/装置（台）		0
软件著作权（项）		1
新工艺（项）		0
新材料（个）		0
研究（咨询）报告（篇）		1
集成电路布图设计专有权（项）		0
培养研究生人数（人）		1

2、 主要研发技术指标

	研发技术指标名称	目标值（含目标单位及数值范围）
1	人工智能化糖尿病管理规范	1套管理规范文书

2	糖尿病高危因素评估	1 份社区糖尿病高危人群筛查报告
3	糖尿病并发症的预测模型	1 套虚拟代谢预测模型
4	社区糖尿病发病报告	1 份报告文书
5	人工智能与医疗大数据的互联互通	完成 1 套信息交换平台

3、 主要成果类指标

	成果指标名称	目标值（含目标单位及数值范围）
1	撰稿论文投稿	4 篇及以上，其中 SCI 文章 1 篇，国内核心期刊 2 篇及以上
2	编制并申报团体标准研究项目	申报 1 项及以上
3	软件著作权	申报 1 项软件著作权

4、 主要经济指标

	经济指标名称	目标值（含目标单位及数值范围）

5、 主要建设任务

	建设任务名称	目标值（含目标单位及数值范围）
1	建立糖尿病综合防控体系科技示范社区	2-3 家科技示范社区

6、 其他考核指标

	其他考核指标名称	目标值（含目标单位及数值范围）

三、项目进度计划及考核指标

阶 段	项目进度计划及考核指标
2020 年 7 月至 2020 年 12 月	制定筛查和管理工作操作手册及人工智能需求分析。
2021 年 [illegible]月至 2021 年 6 月	开发人工智能应用，调整预测模型。

2021年7月至2021年12月	招募研究对象，签署知情同意书，开展筛查和干预管理。
2022年1月至2022年6月	开展筛查和干预管理，调整模型。
2022年7月至2022年12月	开展干预后1年评估，完善干预管理流程，撰写论文并投稿。
2023年1月至2023年6月	整理评估数据，分析筛查和干预管理效果，撰写结题报告。

四、项目承担单位、参加单位及主要研究开发人员

项目承担单位： 苏州市疾病预防控制中心				
项目合作单位	合作单位1名称	苏州米太人工智能研究院有限公司	合作单位1证件号	91320594MA1Q5BFR6X
	合作单位2名称	苏州市卫生计生统计信息中心	合作单位2证件号	12320500466958384B
	合作单位3名称	苏州市姑苏区疾病预防控制中心	合作单位3证件号	123205084669805493
	境外合作单位名称		境外合作单位国家信息	

项目负责人

姓名	性别	年龄	职称/职务	从事专业	为本项目工作时间（XX个月/年）	所在单位
陆艳	女	45	主任医师	慢性病预防与控制	6	苏州市疾病预防控制中心

主要研究开发人员

姓名	性别	出生日期	职称/职务	从事专业	为本项目工作时间（XX个月/年）	所在单位
秦绮玲	男	1956年02月10日	教授	人工智能	3	苏州米太人工智能研究院有限公司
朱杰	男	1981年12月01日	副主任副高	计算机	4	苏州市卫生计生统计信息中心
沈腊梅	女	1988年12月19日	主管医师	慢性病防控	[illegible]	苏州市疾病预防控制中心
张秋	男	1983年11	副主	慢性病防	4	苏州市姑苏区疾病

		月 25 日	任医师	控		预防控制中心
张正姬	女	1979 年 03 月 14 日	副主任医师	慢性病防控	8	苏州市疾病预防控制中心
王临池	女	1985 年 01 月 17 日	副主任医师	慢性病防控	8	苏州市疾病预防控制中心
黄春妍	女	1985 年 03 月 20 日	主管医师	慢性病防控	8	苏州市疾病预防控制中心
韦晓淋	女	1988 年 08 月 25 日	主管医师	慢性病防控	8	苏州市疾病预防控制中心
华钰洁	女	1989 年 11 月 14 日	医师	慢性病防控	8	苏州市疾病预防控制中心
刘亚平	男	1957 年 08 月 25 日	教授	人工智能	3	苏州米太人工智能研究院有限公司
周琦	男	1975 年 08 月 23 日	教授级高工	计算机	4	苏州米太人工智能研究院有限公司
隆彦群	男	1990 年 09 月 08 日	工程师	计算机	5	苏州米太人工智能研究院有限公司
丁翀	男	1984 年 02 月 26 日	工程师	计算机	6	苏州市疾病预防控制中心

五、项目新增经费预算

（一）项目投资规模及建设资金来源与构成比例

单位：万元	合计	备注
项目新增投入经费	31.00	
1、市拨款	30.00	
2、部门、地方配套	1.00	
3、承担单位自筹（含风险投资、银行贷款等）	0.00	
4、其他来源（含合作单位出资等）	0.00	

项目科技经费采取一次性或分年度拨款形式，甲方拨款 30.00 万元，丙方支持 1.00 万元，不足部分由乙方自筹解决。甲方 2020 年核拨乙方首批科技经费 30.00 万元。若项目按计划顺利开展，将于 2021 年，拨款 0.00 万元，2022 年，拨款 0 万元，项目顺利验收后拨款 0.00 万元。

（二）项目建设经费的支出预算

单位：万元	预算数	其中：市财政拨款	备注
新增投入经费合计	31.00	30.00	
（一）直接费用	27.00	27.00	
1、设备费	6.00	6.00	
2、材料费/测试化验加工费/燃料动力费	9.00	9.00	
3、差旅费/会议费/国际合作与交流费	6.00	6.00	
4、劳务费/专家咨询费	3.00	3.00	
5、其他支出	3.00	3.00	
（二）间接费用	4.00	3.00	
1、绩效支出	3.00	3.00	
2、管理费	1.00	0.00	

说明：

项目经费由直接费用和间接费用组成，编制科目预算时只需测算总额。

（一）直接费用是指在项目实施过程中发生的与之直接相关的费用。主要包括：

1、设备费：是指项目实施过程中购置或试制专用仪器设备，对现有仪器设备进行升级改造，以及租赁外单位仪器设备而发生的费用。应当严格控制设备购置，鼓励开放共享、自主研制、租赁专用仪器设备以及对现有仪器设备进行升级改造，避免重复购置。

2、材料费/测试化验加工费/燃料动力费：

材料费是指在项目实施过程中消耗的各种原材料、辅助材料等低值易耗品的采购及运输、装卸、整理等费用。

测试化验加工费是指在项目实施过程中支付给外单位（包括项目承担单位内部独立经济核算单位）的检验、测试、化验及加工等费用。

燃料动力费是指在项目实施过程中直接使用的相关仪器设备、科学装置等运行发生的水、电、气、燃料消耗费用等。

3、差旅费/会议费/国际合作与交流费：

差旅费是指在项目实施过程中开展科学实验（试验）、科学考察、业务调研、学术交流等所发生的外埠差旅费、市内交通费用等。差旅费的开支标准应当按照国家、省、市有关规定执行。

会议费是指在项目实施过程中为了组织开展或参加学术研讨、咨询以及协调项目开展工作等活动而发生的会议费用。会议费支出应当按照国家、省、市有关规定执行。

国际合作与交流费是指在项目实施过程中项目研究人员出国及赴港澳台、外国专家来华及港澳台专家来内地工作的费用。国际合作与交流费应当严格执行国家、省、市外事资

金管理的有关规定。

承担单位和科研人员应当按照实事求是、精简高效、厉行节约的原则，统筹安排使用。

4、劳务费/专家咨询费：

劳务费是指在项目实施过程中支付给参与项目的研究生、博士后、访问学者以及项目聘用的研究人员、科研辅助人员等的劳务性费用。

项目聘用人员的劳务费开支标准，参照当地科学研究和技术服务业从业人员平均工资水平，根据其在项目研究中承担的工作任务确定，其社会保险补助纳入劳务费科目开支。劳务费预算应据实编制，不设比例限制。

专家咨询费是指在项目实施过程中支付给临时聘请的咨询专家的费用。专家咨询费不得支付给参与项目及所属课题研究和管理的相关工作人员。

5、其他支出：是指在项目实施过程中除上述支出范围之外的其他相关支出。

（二）间接费用是指项目实施单位在组织实施项目过程中发生的无法在直接费用中列支的相关费用，包括有关管理费和绩效支出等。

提高项目间接费用比例，按照不超过直接费用扣除设备费后的一定比例核定，具体比例为：500 万元及以下部分为 30%，500 万元至 1000 万元的部分为 25%，1000 万元以上的部分为 20%。

间接费用由实施单位统筹安排使用。实施单位应当建立健全间接费用的内部管理办法，公开透明、合规合理使用间接费用，处理好分摊间接成本和对科研人员激励的关系。绩效支出安排应当与科研人员在项目工作中的实际贡献挂钩。绩效支出不设比例限制。间接费用的绩效支出不计入项目承担单位绩效工资总额基数，纳入项目承担单位绩效工资总量管理。间接费用的绩效支出中，给予 35 周岁以下青年科技人员的比例原则上不低于 30%。

（三）项目实施的组织管理措施和项目资金的管理办法：

本项目将严格按照《苏州市科技计划项目管理办法》（苏科规〔2019〕2 号）、《苏州市市级科技创新专项资金管理办法》（苏财规〔2017〕6 号）、《苏州市科技信用管理办法(试行)》（苏科规〔2015〕3 号）等有关规定实施项目组织管理措施、合理使用项目资金。

六、项目管理依据

项目管理严格按照《苏州市科技计划项目管理办法》（苏科规〔2019〕2 号）、《苏州市市级科技创新专项资金管理办法》（苏财规〔2017〕6 号）、《苏州市科技计划项目信用管理办法》（苏科规〔2020〕[illegible]号）等有关规定执行。

七、共同条款

1、合同签订后，乙方必须落实项目管理的有关要求，丙方承担监督和协助的职责，加强项目实施和资金使用的日常跟踪管理。在此基础上，甲方和丙方协助和督促所辖财政局及时拨款。申请项目明确需要地方财政支持资金的，丙方应及时和财政部门联系，协调安排落实资金。

2、乙方应及时落实项目自筹经费，保障项目顺利实施。对市科技项目经费单独建账、专项核算、专款专用。项目经费不得用于支付各种罚款、捐款、赞助、投资等支出，不得用于国家规定禁止列入的其他支出。

3、在项目总预算不变的情况下，项目负责人可根据科研活动实际需要，在预算范围内自主安排经费开支，自主调整直接费用全部科目的经费支出，不受比例限制，由乙方办理调剂手续，并可作为项目验收、评估或审计检查的依据。如因项目建设预算控制不严，发生经费超支，由乙方自筹解决，不得影响项目的时序进度和完工期限。

4、项目负责人对项目实施具有自主选择和调整技术路线的自主权。在不降低研究目标的前提下，项目负责人可以按照研发创新规律和市场环境变化自主调整研究方案和技术路线，报乙方备案。项目实施期内，项目负责人可按规定自主组建科研团队，并结合项目实施进展情况进行相应调整。上述安排和调整均可作为项目验收、评估评审和审计检查等的依据。

5、项目如发生项目负责人或承担单位变更、项目主要研究目标或考核指标调整，承担单位须及时提出书面报告，经丙方审查同意后，由丙方报甲方。

6、乙方应在项目合同约定执行期到期后一年内完成验收，提前完成的项目可以提前申请组织验收。因客观原因不能在规定期限内完成研究任务且需延长项目实施期限的项目，乙方应在合同规定的项目实施期结束前提出延期申请，经丙方审查同意后向甲方报备。实施期限最长延期一年，报备文件可作为项目合同书的补充材料及项目后续实施、管理的依据。**项目到期后不可调整。**

7、乙方应按要求及时报送项目执行情况和有关统计报表，按有关财务规定妥善保存有关原始票据及凭证备查，做好相关台帐管理，在单位会计核算系统中单独设置会计科目或设辅助明细账。不能作为“单独核算”的方式主

动配合甲方和丙方的监督检查，并按要求提供相关文件资料。

8、项目在实施过程中因客观原因或不可抗拒因素导致项目无法正常实施的，乙方可主动提出终止项目的申请，附已做工作的书面总结，按规定对项目经费进行审计，且经丙方审核同意后报甲方。对已经勤勉尽责、未谋私利，但因技术路线选择失误或其他不可预见原因，导致未完成市科技项目任务目标的单位和负责人予以免责，不记入信用记录。

9、乙方因主观原因发生严重科研不端行为、拒不开展项目实质性研发、项目经费使用故意违规等原因导致项目无法正常实施的，以及在项目合同约定执行期到期后18个月内未完成验收的，由丙方提出申请，经甲方审核后，予以强制终止并记入科研信用记录。甲方有权对乙方的失信行为进行记录并对外公布。

10、乙方若有项目合作单位，须与合作单位签订针对本项目的非框架性的具体合作协议，并明确项目成果的归属，对研究内容、经费的分配及安排、成果产权的归属均予以明确。非归属乙方及合作单位的成果不能算入本项目的产出。乙方有督促合作单位开展工作的义务。

11、丙方（地方科技主管部门）应将本项目列入本地或本系统的科技发展计划进行管理，监督检查并保证合同条款的执行，协助解决合同执行过程中出现的问题。市本级行政、事业单位所承担科技计划项目的财务决算和监督，丙方（主管局）扎口管理。

12、甲、乙、丙各方对技术经济资料负有保密责任。

13、本合同文本一式四份，其中乙方执两份，甲、丙双方各执一份。

苏州科技

签订合同、责任书各方：

甲方：

科技局法定代表人委托代理人（签字）：　　公　章

处室负责人（签字）：沈丹

年　月　日

2020年11月9日

乙方：

法定代表人或委托代理人（签字）：　　公　章

课题负责人（签字）：陆艳

2020年11月6日

开户银行、帐号：

丙方：

法定代表人或委托代理人（签字）：　　公　章

委托代理人（签字）：

年　月　日

苏州科技
